# HCAR2 Modulates the Crosstalk between Mammary Epithelial Cells and Macrophages to Mitigate *Staphylococcus aureus* Infection in the Mouse Mammary Gland

**DOI:** 10.1002/advs.202411947

**Published:** 2025-01-10

**Authors:** Xin Ran, Kefei Li, Yutao Li, Weiwei Guo, Xiaoxuan Wang, Wenjin Guo, Bao Yuan, Juxiong Liu, Shoupeng Fu

**Affiliations:** ^1^ State Key Laboratory for Diagnosis and Treatment of Severe Zoonotic Infectious Diseases Key Laboratory for Zoonosis Research of the Ministry of Education Institute of Zoonosis College of Veterinary Medicine Jilin University Changchun Jilin 130062 China; ^2^ Department of Laboratory Animals College of Animal Sciences Jilin Provincial Key Laboratory of Animal Model Jilin University Changchun Jilin 130062 China

**Keywords:** CMPK2, HCAR2, mastitis, pyroptosis, S. aureus

## Abstract

*Staphylococcus aureus* (*S. aureus*) is a major zoonotic pathogen, with mammary gland infections contributing to mastitis, a condition that poses significant health risks to lactating women and adversely affects the dairy industry. Therefore, understanding the immune mechanisms underlying mammary infections caused by *S. aureus* is essential for developing targeted therapeutic strategies against mastitis. This study identified hydroxycarboxylic acid receptor 2 (HCAR2) as a potential regulator of *S. aureus* infection in mammary glands. It is demonstrated that HCAR2 deficiency exacerbates the inflammatory response and disrupts the blood‐milk barrier in the mammary gland during *S. aureus* infection, with NLRP3 inflammasome‐mediated pyroptosis playing a central role. Activation of HCAR2, on the other hand, suppressed CMPK2 expression, thereby mitigating mitochondrial damage and pyroptosis in mouse mammary epithelial cells (mMECs) induced by *S. aureus*. Additionally, mitochondrial DNA (mtDNA) released from *S. aureus*‐infected mMECs activates the cGAS/STING signaling pathway in macrophages, impairing their bactericidal activity. In conclusion, this study highlights the critical role of HCAR2 in *S. aureus* infection of the mammary gland and provides a theoretical basis for identifying potential therapeutic targets for such infections.

## Graphical abstract

In the mammary gland infected with *S. aureus*, the activation of hydroxycarboxylic acid receptor 2 (HCAR2) in mammary epithelial cells (mMECs) leads to a reduction in CMPK2 expression, which in turn mitigates cell pyroptosis. The attenuation of pyroptosis in mMECs reduces the release of mitochondrial DNA (mtDNA) into the extracellular space, thereby alleviating the suppression of macrophage activity induced by *S. aureus* via the cGAS/STING‐dependent pathway.

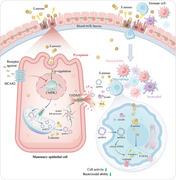

## 1 Introduction

*Staphylococcus aureus* (*S. aureus*) is a major zoonotic pathogen responsible for extensive clinical infections in both humans and livestock, posing a significant threat to public health due to its infectious nature and pathogenic potential.^[^ ^1^, ^2^ ^]^ Mammary gland infections caused by *S. aureus* are a primary contributor to mastitis, often progressing to purulent mastitis, which severely disrupts the structural integrity of the mammary gland. If not promptly and effectively treated, mastitis can lead to systemic infectious complications, endangering the health of lactating women and negatively impacting the dairy industry.^[^ ^3^, ^4^, ^5^ ^]^ Currently, *S. aureus* exhibits resistance to many commonly used antibiotics, and even strains classified as sensitive can still cause severe infections in the host.^[^ ^6^, ^7^ ^]^ Therefore, understanding the immune mechanisms involved in *S. aureus*‐induced host infections and developing strategies to bolster the host's defense mechanisms is critical. This approach is essential for preventing further resistance development and minimizing the impact of broad‐spectrum antibiotics on beneficial microbiota, thereby enabling more effective targeted treatment strategies for managing infections.

The innate immune system plays a pivotal role in the early detection and elimination of invading *S. aureus*, utilizing pattern recognition receptors (PRRs) to identify pathogen‐associated molecular patterns (PAMPs) and initiate appropriate defense responses within host cells.^[^ ^8^ ^]^ Mammary epithelial cells (mMECs), as the functional units of the mammary gland, are primarily responsible for synthesizing, metabolizing, and maintaining the integrity of the blood‐milk barrier. Loss of functionality in these cells can lead to irreversible disruption of the blood‐milk barrier.^[^ ^9^, ^10^ ^]^ During mammary infection, these epithelial cells act as sentinel cells, initially detecting components of *S. aureus* and contributing to the host's defense mechanisms. However, *S. aureus* can induce various forms of programmed cell death (PCD) in mMECs, including pyroptosis, a highly inflammatory type of cell death. This mode of cell death not only compromises the blood‐milk barrier but also facilitates the recruitment of diverse immune cell populations.^[^ ^11^, ^12^ ^]^ Although recruited macrophages and neutrophils are essential for pathogen clearance and inflammatory response regulation, their excessive pro‐inflammatory activation is a key factor in mammary tissue damage.^[^ ^13^ ^]^ Studies suggest that excessive epithelial cell death can trigger a dysregulated inflammatory response, impairing the functionality of immune cells, including macrophages.^[^ ^14^ ^]^ Upon cell death, damage‐associated molecular patterns (DAMPs) and cellular debris are released, which are subsequently engulfed and degraded by macrophages through phagocytosis. However, excessive cell death, along with varying modes of PCD, can directly affect the functional capacity and efficiency of macrophages.^[^ ^15^, ^16^ ^]^ Consequently, host responses during pathogen elimination may lead to detrimental inflammation, exacerbating clinical symptoms. A comprehensive understanding of the innate immune mechanisms employed during *S. aureus* mammary infections is essential for identifying novel prevention and treatment strategies.

G protein‐coupled receptors (GPCRs) are integral to various physiological processes, including immune regulation. Endogenous peptides, short‐chain fatty acids, and proteases serve as natural ligands for GPCRs.^[^ ^17^ ^]^ This study identifies hydroxycarboxylic acid receptor 2 (HCAR2) as a key player in *S. aureus* infection within mammary tissue. HCAR2 is expressed in mMECs and macrophages, with endogenous compounds such as nicotinic acid (Nia) acting as ligands for HCAR2.^[^ ^18^, ^19^ ^]^ Additionally, research by Guo et al. demonstrated that HCAR2 activation can mitigate sepsis, highlighting its role in infectious diseases.^[^ ^20^ ^]^ Despite this, the precise function and underlying mechanisms of HCAR2 in *S. aureus* infection remain unclear. This study elucidates the role of HCAR2 in modulating *S. aureus* infection in the mammary gland and provides novel insights into potential therapeutic targets for *S. aureus*‐related diseases. These findings offer significant scientific value in developing strategies to reduce the incidence and progression of such infections.

## 2 Results

### 2.1 HCAR2 Exhibits a Significant Correlation with Mammary Infections caused by *S. aureus*

**Figure 1:**
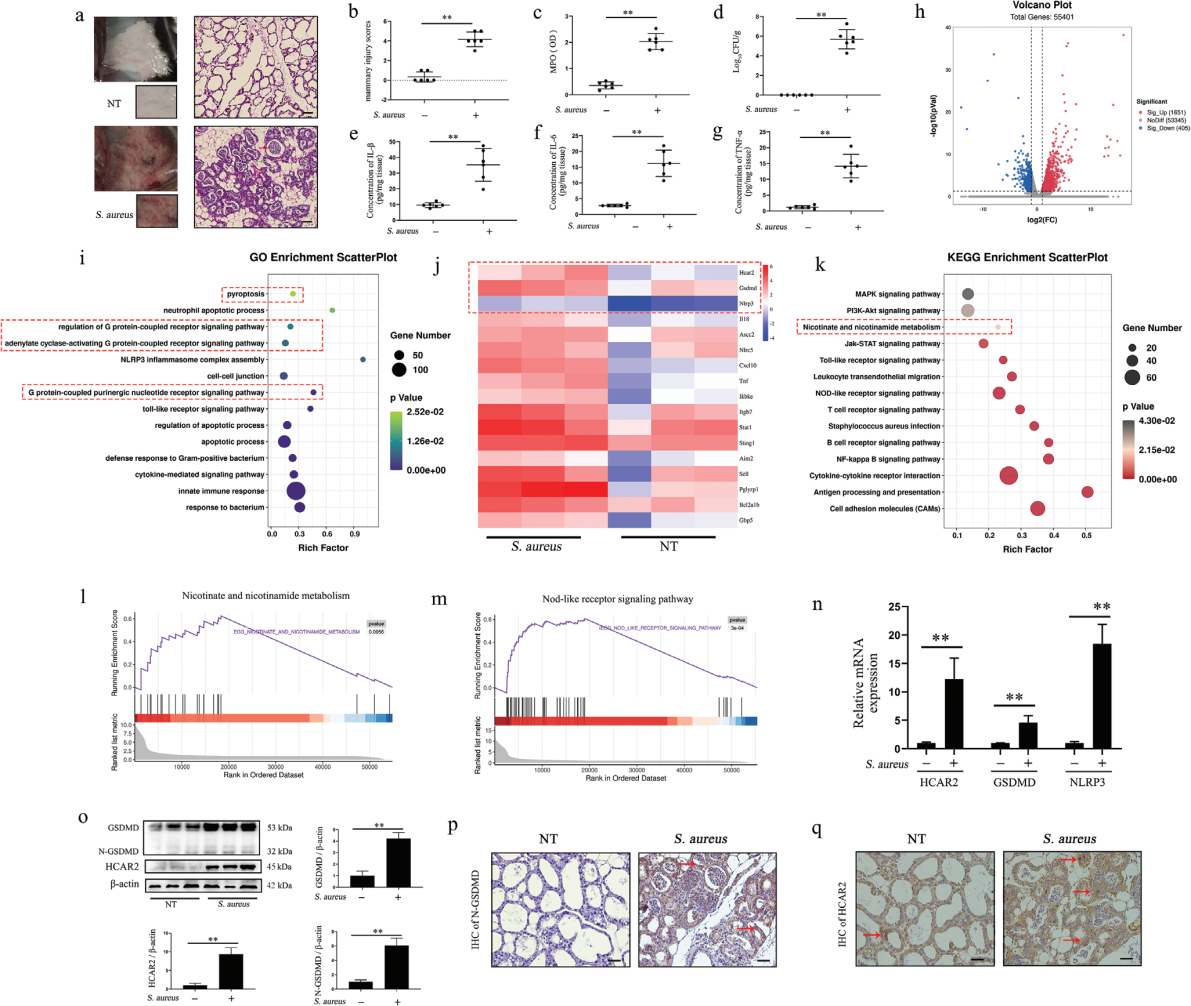
HCAR2 exhibits a significant correlation with mammary infections caused by *S. aureus*. a) Images of mouse mammary glands and H&E staining; red arrows indicate inflammatory cell infiltration, green arrows highlight thickened acinar walls, and Scale bar represents 100 µm. b) Mammary injury score. c) MPO content in mammary tissue. d) Bacterial burden in mammary tissue. e–g) ELISA quantification of IL‐1β, IL‐6, and TNF‐α protein levels in mammary tissue. h) Transcriptome volcano plot of mammary gland tissues. “NoDiff” represents no significant difference in gene expression between groups, “Sig‐Up” indicates upregulation compared to the NT group, and “Sig‐Down” indicates downregulation compared to the NT group. i) GO enrichment analysis. j) Cluster analysis of differentially expressed genes. k) KEGG enrichment analysis. l, m) GSEA analysis for Nicotinate and Nicotinamide metabolism and NOD‐like receptor signaling pathway. n) mRNA expression of HCAR2, NLRP3, and GSDMD in mammary tissues. o) Western blot analysis of GSDMD and HCAR2 protein levels in mammary tissues. p, q) Immunohistochemistry (IHC) staining for N‐GSDMD and HCAR2 in mouse mammary tissue. Scale bar = 50 µm. Results are presented as mean ± SD, n = 6.

To identify the key target involved in *S. aureus*‐induced mammary infection, a mouse model of mastitis was established using *S. aureus*. Infection with *S. aureus* resulted in significant swelling and redness of the mammary glands, alveolar degeneration, and inflammatory cell infiltration within the alveolar cavity (**Figure** **1**a,b). Furthermore, *S. aureus* infection led to a marked increase in MPO content, bacterial load, and pro‐inflammatory factors within the mammary gland (Figure 1c–g). Transcriptome sequencing was performed to analyze gene expression profiles in the mammary tissues of the NT and *S. aureus* groups. The analysis revealed a total of 2056 differentially expressed genes between the two groups (Figure 1h). Gene Ontology (GO) enrichment analysis indicated significant differences in several pathways, including innate immune response, pyroptosis, and various pathways related to GPCRs (Figure 1i). Additionally, the Kyoto Encyclopedia of Genes and Genomes (KEGG) pathway enrichment analysis identified alterations in nicotinic acid metabolism. Further clustering of differentially expressed genes revealed a significant upregulation of the G protein‐coupled receptor HCAR2, activated by Nia, in the *S. aureus*‐stimulated group (Figure 1j,k). Gene set enrichment analysis (GSEA) further highlighted the upregulation of the NOD‐like receptor signaling pathway and alterations in nicotinic acid and nicotinamide metabolism in mammary tissue following *S. aureus* stimulation (Figure 1l,m). To validate the results of the transcriptome sequencing, HCAR2 expression was examined. The results confirmed that *S. aureus* stimulation significantly induced the transcription and translation of GSDMD and HCAR2 in mammary tissue, with immunohistochemistry (IHC) results consistent with the protein expression data (Figure 1n–q). These results suggest that HCAR2 is involved in mammary infections induced by *S. aureus*.

### 2.2 HCAR2 Deficiency Exacerbates the Severity of *S. aureus* Infection in Mouse Mammary Glands

**Figure 2:**
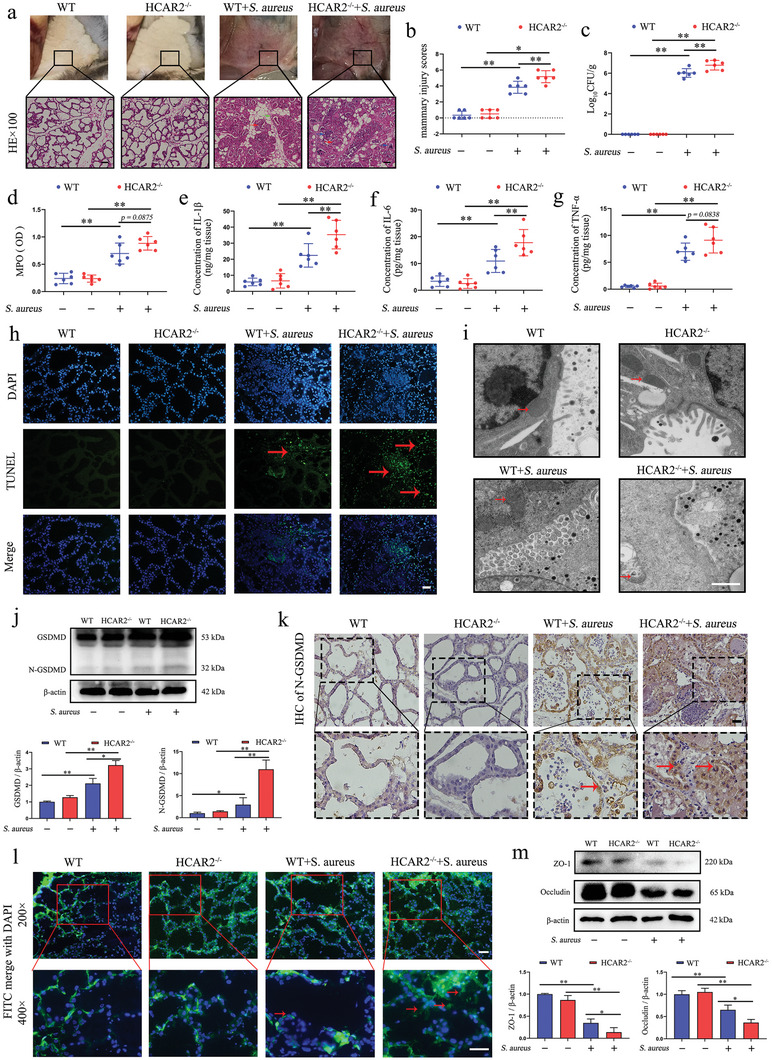
HCAR2 deficiency exacerbates the severity of *S. aureus* infection in mouse mammary glands. a) Images of mouse mammary glands and H&E staining; red arrows indicate inflammatory cell infiltration, green arrows highlight thickened acinar walls, blue arrows point to tissue necrosis, and the scale bar represents 100 µm. b) Mammary injury scores. c) Bacterial burden in mammary tissue. d) MPO content in mammary tissue. e–g) ELISA quantification of IL‐1β, IL‐6, and TNF‐α protein levels in mammary tissue. h) TUNEL staining of mammary glands; red arrows mark TUNEL‐positive cells, scale bar represents 50 µm. i) Transmission electron microscopy of mitochondrial integrity; red arrows indicate mitochondria, and the scale bar represents 2 µm. j) Western blot analysis of GSDMD protein expression in mammary tissue. k) IHC of N‐GSDMD in mammary tissue, scale bar represents 50 µm. l) FITC‐albumin fluorescence in mammary tissue; green fluorescence indicates FITC‐albumin, blue fluorescence marks nuclei, and red arrows indicate FITC‐albumin diffusion in acini, scale bar represents 50 µm. m) Western blot analysis of ZO‐1 and Occludin protein expression in mammary tissue. Results are presented as mean ± SD, n = 6.

To elucidate the role of HCAR2 in *S. aureus* infection of the mammary gland, experiments were conducted using HCAR2 knockout (HCAR2^−/−^) mice. Initial confirmation of HCAR2 deletion in the mammary glands of these mice was performed (Figure S1, Supporting Information). Subsequently, a mastitis model was established in both wild‐type (WT) and HCAR2^−/−^ mice by infecting them with *S. aureus*. No noticeable differences were observed between the mammary glands of WT and HCAR2^−/−^ mice under baseline conditions. However, after infection, HCAR2^−/−^ mice exhibited significantly more pronounced redness and swelling in the mammary glands compared to the NT group, along with an increased number of necrotic foci within the acini. This resulted in a higher tissue damage score for HCAR2^−/−^ mice than for WT mice (**Figure** **2**a,b). Furthermore, HCAR2^−/−^ mice showed a higher bacterial load in the mammary glands, although no significant difference in MPO content was observed between HCAR2^−/−^ and WT mice (Figure 2c,d). Elevated levels of IL‐1β and IL‐6 were also detected in the mammary glands of HCAR2^−/−^ mice compared to WT mice (Figure 2e–g). These results suggest that HCAR2 deficiency exacerbates *S. aureus* infection and induces a more intense inflammatory response in the mammary gland.

Additionally, *S. aureus* infection led to an increase in cell death in the mammary glands of both WT and HCAR2^−/−^ mice. Notably, the number of dead cells was significantly higher in HCAR2^−/−^ mice than in WT mice (Figure 2h). Given the close association between cell death and mitochondrial damage, mitochondrial integrity in mMECs was assessed. Results revealed significantly more severe mitochondrial damage in HCAR2^−/−^ mice compared to WT mice (Figure 2i). *S. aureus* is known to induce pyroptosis, exacerbating tissue damage.^[^ ^21^ ^]^ Our results showed that *S. aureus* infection led to a marked increase in GSDMD activation in the mammary glands of both WT and HCAR2^−/−^ mice, with the latter, showing a more pronounced effect (Figure 2j,k). The blood‐milk barrier plays a critical role in maintaining intramammary stability, and epithelial cell pyroptosis accelerates its disruption. FITC‐albumin diffusion assays revealed that *S. aureus* infection induced albumin leakage in the mammary glands of both WT and HCAR2^−/−^ mice. However, the leakage was significantly more pronounced in HCAR2^−/−^ mice than in WT mice (Figure 2l). Additionally, *S. aureus* infection caused a reduction in tight junction (TJ) protein expression, with lower levels of these proteins observed in HCAR2^−/−^ mice compared to WT mice (Figure 2m). These results indicate that HCAR2 deficiency exacerbates pyroptosis and the disruption of the blood‐milk barrier during *S. aureus* infection in the mammary gland.

### 2.3 HCAR2 Deficiency enhances NLRP3 Inflammasome Activation in Mammary Glands of *S. aureus*‐Infected Mice

**Figure 3:**
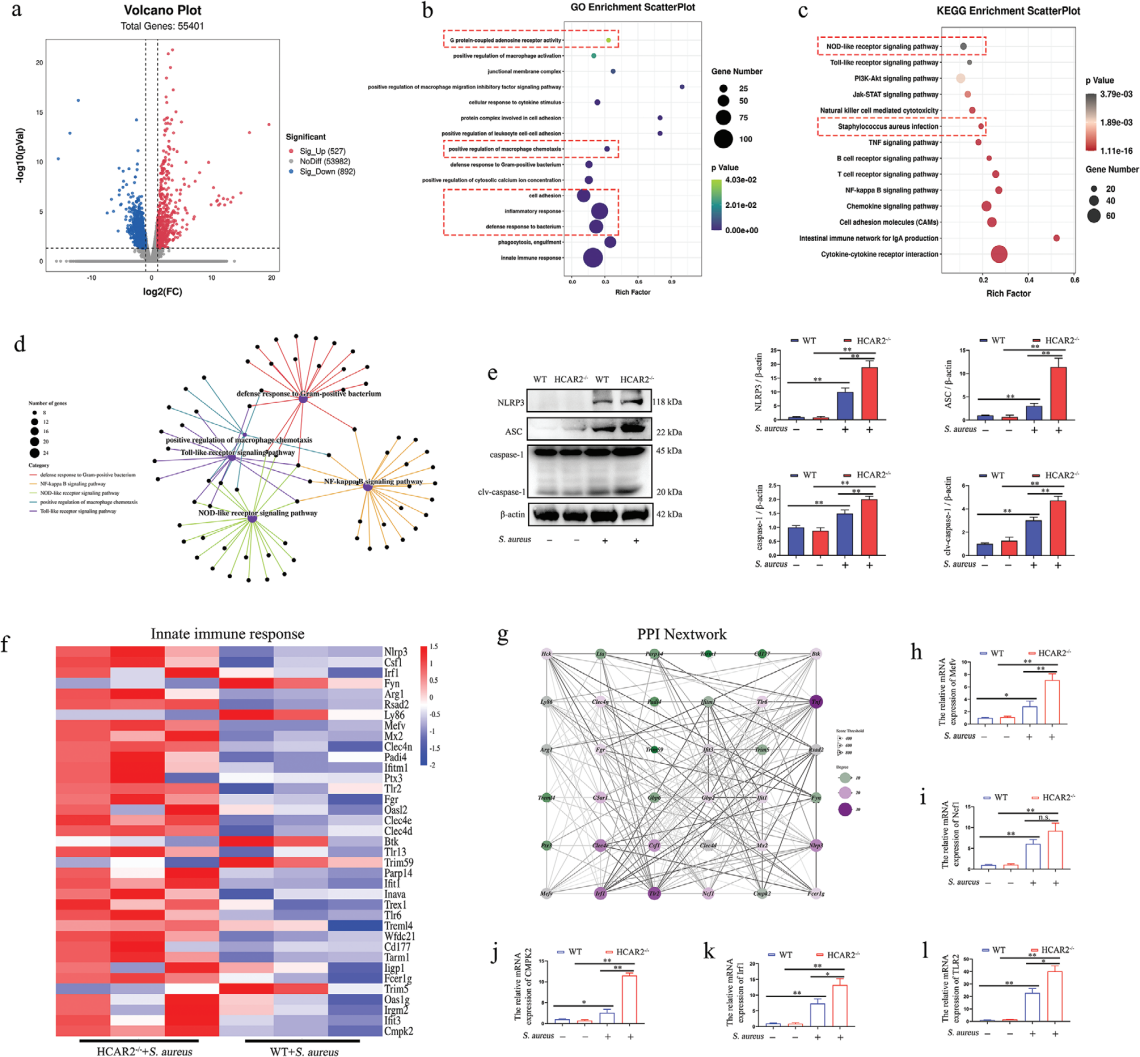
HCAR2 deficiency enhances NLRP3 inflammasome activation in mammary glands of *S. aureus*‐infected mice. a) Transcriptome volcano plot of mammary gland tissues. “NoDiff” indicates no significant difference in gene expression between the two groups, while “Sig‐Up” and “Sig‐Down” represent upregulated and downregulated genes, respectively, in comparison to the NT group (b) GO enrichment analysis. c) KEGG enrichment analysis. d) Gene‐pathway enrichment network diagram. e) Western blot detection of NLRP3, ASC, and caspase‐1 protein levels in mammary tissue. f) Cluster analysis of differential genes associated with innate immune response. g) Protein‐protein interaction (PPI) analysis. h–l) mRNA expression levels of Mefv, Ncf1, CMPK2, Irf1, and TLR2 in mammary tissues. Results are presented as mean ± SD, n = 3.

To further explore the regulatory mechanisms of HCAR2 in *S. aureus*‐induced mammary gland infection, transcriptomic analysis was performed to compare gene expression changes in the mammary glands of WT and HCAR2^−/−^ mice following *S. aureus* infection. The analysis revealed 527 upregulated and 892 downregulated genes in the mammary glands of HCAR2^−/−^ mice compared to WT mice (**Figure** **3**a). GO enrichment analysis indicated that these differential genes are involved in bacterial defense responses, macrophage chemotaxis, and activation of GPCRs (Figure 3b). KEGG enrichment analysis highlighted significant differences in pathways such as *S. aureus* infection, Toll‐like receptor signaling, and NOD‐like receptor signaling (Figure 3c). The gene‐pathway enrichment network diagram demonstrated a close association between the Gram‐positive bacterial defense response, positive regulation of macrophage chemotaxis, and the NF‐κB signaling pathway with the NOD‐like receptor signaling pathway (Figure 3d). Given the pivotal role of the NOD‐like receptor signaling pathway in regulating pyroptosis, the activation of the NLRP3 inflammasome was assessed. The results showed that in HCAR2^−/−^ mice, *S. aureus* stimulation led to significantly higher activation of the NLRP3 inflammasome compared to WT mice (Figure 3e). Clustering analysis of differential genes related to innate immune responses revealed significantly higher expression levels of NLRP3, TLR2, and Mefv in the mammary glands of HCAR2^−/−^ mice than in WT mice (Figure 3f). Furthermore, this study identified genes involved in regulating the NLRP3 inflammasome and validated several candidates (Figure 3g). The results demonstrated that in *S. aureus*‐infected HCAR2^−/−^ mice, the mRNA expression levels of CMPK2, Mefv, Irf1, and TLR2 were significantly elevated compared to those in WT mice (Figure 3h–l).

### 2.4 Nia exerts a Significant Alleviating Effect on *S. aureus* Infection in the Mammary Glands of Mice

**Figure 4:**
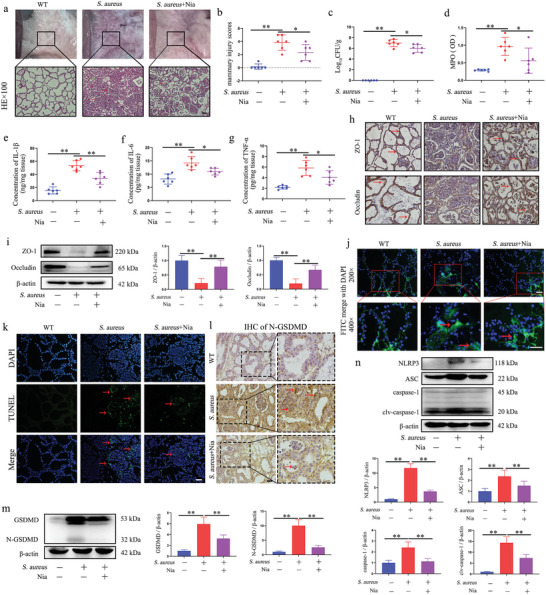
Nia has a significant alleviating effect on *S. aureus* infection in the mammary glands of mice. a) Images of mouse mammary glands and H&E staining; red arrows indicate inflammatory cell infiltration, green arrows highlight thickened acinar walls, blue arrows point to tissue necrosis, and the scale bar represents 100 µm. b) Mammary injury score. c) Bacterial burden in mammary tissue. d) MPO content in mammary tissue. e–g) ELISA detection of IL‐1β, IL‐6, and TNF‐α protein levels in mammary tissue. h) IHC of ZO‐1 and Occludin in mammary tissue, scale bar represents 50 µm. i) Western blot detection of ZO‐1 and Occludin protein expression in mammary tissue. j) FITC‐albumin fluorescence in mammary tissue, with green fluorescence representing FITC‐albumin, blue fluorescence representing the nucleus, and red arrows indicating FITC‐albumin diffusion in the acini, scale bar representing 50 µm. k) TUNEL staining in the mammary gland, with red arrows indicating TUNEL‐positive cells, the scale bar represents 50 µm. l) IHC of N‐GSDMD in mammary tissue, scale bar represents 50 µm. n) Western blot detection of NLRP3, ASC, and caspase‐1 protein expression in mammary tissue. m) Western blot detection of ZO‐1, Occludin, and Claudin 3 protein expression in mammary tissue. Results are presented as mean ± SD, n = 6.

Based on the results above, the potential alleviating effect of HCAR2 activation on *S. aureus*‐induced mastitis was explored. Nia treatment significantly ameliorated the pathological changes in mammary gland tissue induced by *S. aureus* stimulation (**Figure** **4**a,b). Moreover, Nia treatment led to a marked reduction in MPO content, bacterial burden, and pro‐inflammatory cytokine levels in the mammary tissue (Figure 4c–g). Additionally, Nia alleviated the downregulation of TJ proteins caused by *S. aureus* infection in the mammary glands (Figure 4h,i). Furthermore, Nia treatment notably reduced FITC‐albumin diffusion within the mammary ducts, a hallmark of barrier disruption induced by *S. aureus* (Figure 4j). Further analysis revealed that Nia inhibited the increase in dead cells triggered by *S. aureus* infection in the mammary gland (Figure 4k). Nia also significantly decreased the up‐regulation of N‐GSDMD expression in mammary tissue induced by *S. aureus* (Figure 4l,m). Furthermore, Nia treatment inhibited the activation and initiation of the NLRP3 inflammasome induced by *S. aureus* in the mammary glands (Figure 4n). These results suggest that HCAR2 activation, through Nia, has a substantial alleviating effect on *S. aureus* infection in the mammary glands of mice.

### 2.5 Activation of HCAR2 Mitigates Pyroptosis and Tight Junction Damage Induced by *S. aureus* in mMECs

**Figure 5:**
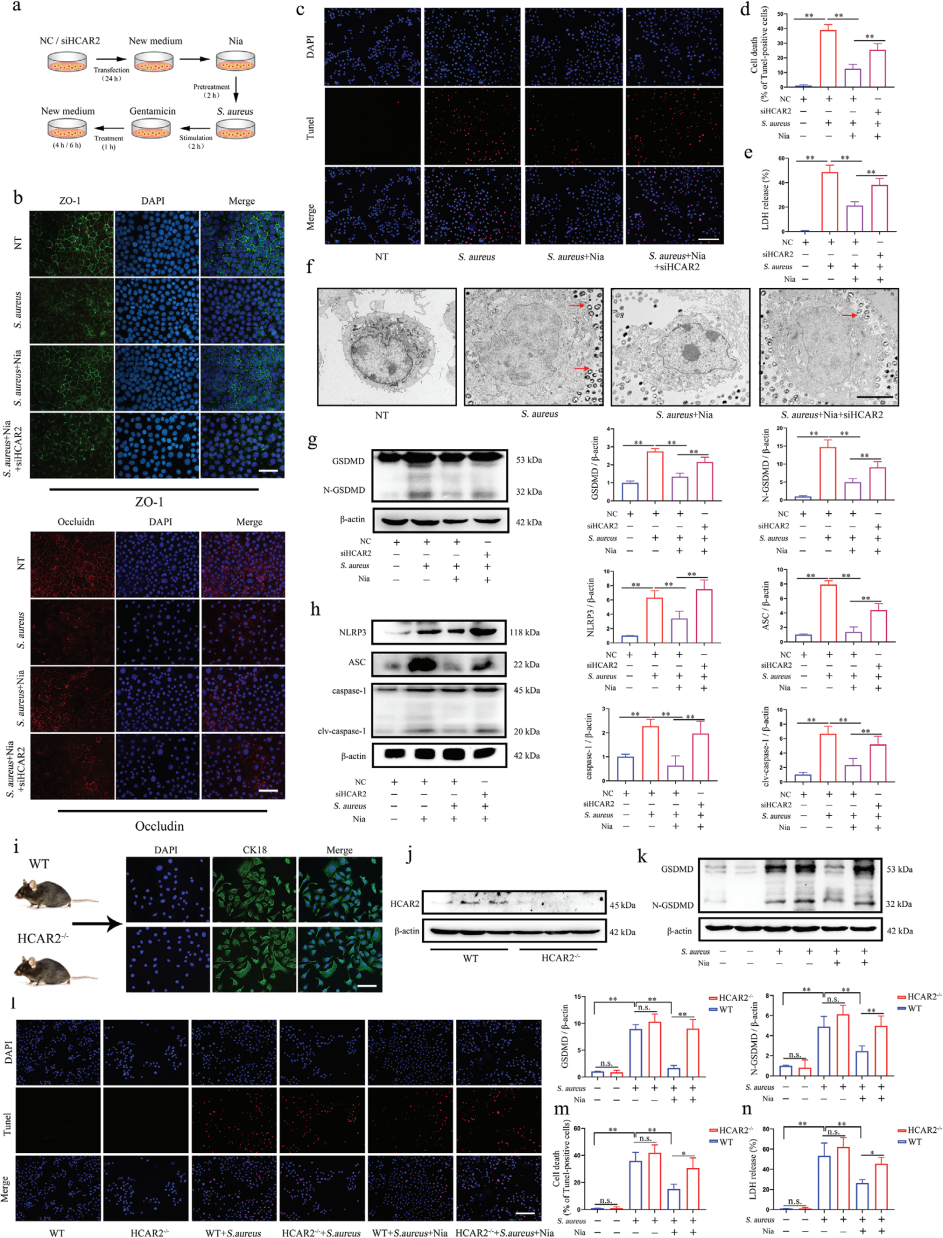
Activation of HCAR2 mitigates pyroptosis and tight junction damage induced by S. aureus in mMECs. a) Schematic representation of the *S. aureus* infection model in vitro. b) Immunofluorescence images of ZO‐1 and Occludin in mMECs, with scale bar representing 100 µm. c) TUNEL staining of mMECs, with red fluorescence indicating TUNEL‐positive cells, scale bar represents 200 µm. d) Quantitative analysis of TUNEL‐positive cells. e) LDH release assay to measure LDH levels in mMECs. f) Transmission electron microscopy (TEM) images showing cellular morphology of mMECs, with red arrows indicating mitochondria and blue arrows indicating cell membranes. The scale bar represents 5 µm. g) Western blot analysis of GSDMD expression in mMECs. h) Western blot analysis of NLRP3, ASC, and caspase‐1 expression in mMECs. i) Immunofluorescence staining for CK18 in primary mammary epithelial cells, with scale bar representing 100 µm. j) Western blot analysis of HCAR2 expression in primary mMECs. k) Western blot analysis of GSDMD expression in primary mMECs. l) TUNEL staining of primary mMECs, with red fluorescence indicating TUNEL‐positive cells, scale bar represents 200 µm. m) Quantitative analysis of TUNEL‐positive cells in mMECs. n) LDH release assay to measure LDH levels in primary mMECs. Results are presented as mean ± SD, n = 3.

The mMECs, neutrophils, and macrophages interact to maintain immune homeostasis within the mammary gland. To investigate how HCAR2 regulates cell‐cell interactions and influences the mammary immune response, *S. aureus* was injected into the mammary glands of WT and HCAR2^−/−^ mice at various time points. The results revealed that, before the substantial recruitment of immune cells, mMECs in HCAR2^−/−^ mice underwent pyroptosis earlier than those in WT mice (Figure S2, Supporting Information). To further examine the role of HCAR2 in mMECs infected with *S. aureus*, Nia and siHCAR2 were used to activate or knock down HCAR2, respectively (**Figures** 1, 5). Nia treatment inhibited the upregulation of pro‐inflammatory factors induced by *S. aureus* through HCAR2 (Figure S3a–c, Supporting Information). Additionally, Nia enhanced the expression of TJ proteins in *S. aureus*‐infected mMECs via HCAR2 (Figure 5b; Figure S3d,e, Supporting Information), suggesting that HCAR2 activation mitigates the inflammatory response and TJ disruption induced by *S. aureus* in mMECs.

In vitro TUNEL staining results showed that Nia reduced cell death induced by *S. aureus* through HCAR2 activation. Furthermore, Nia inhibited the release of LDH from mMECs following *S. aureus* infection via HCAR2 (Figure 5c–e). Morphological analysis of the cells revealed that *S. aureus* infection induced swelling and bubble‐like protrusions in mMECs, which were significantly ameliorated by HCAR2 activation (Figure 5f). GSDMD expression and activation were significantly increased in mMECs upon *S. aureus* stimulation, and Nia reduced GSDMD expression and activation through HCAR2 (Figure 5g). Additionally, Nia significantly decreased the expression of NLRP3, ASC, caspase‐1, and cleaved caspase‐1 in mMECs through HCAR2 (Figure 5h). Co‐localization analysis showed that *S. aureus* stimulation induced co‐localization of ASC and caspase‐1 in mMECs, whereas Nia inhibited this co‐localization via HCAR2 (Figure S3f, Supporting Information). Finally, primary mMECs isolated from WT and HCAR2^−/−^ mice were characterized (Figure 5i,j). It was demonstrated that Nia also alleviated *S. aureus*‐induced pyroptosis in primary mMECs through HCAR2 activation (Figure 5k–n). The detection of pro‐inflammatory factors, tight junctions, and NLRP3 inflammasome components in primary mMECs confirmed the results observed in previous experiments (Figure S4a–h, Supporting Information). These results further support that HCAR2 activation mitigates pyroptosis and tight junction damage induced by *S. aureus* in mMECs.

### 2.6 HCAR2 Inhibits Pyroptosis and Tight Junction Damage in mMECs Induced by S. aureus through Downregulation of CMPK2 Expression

**Figure 6:**
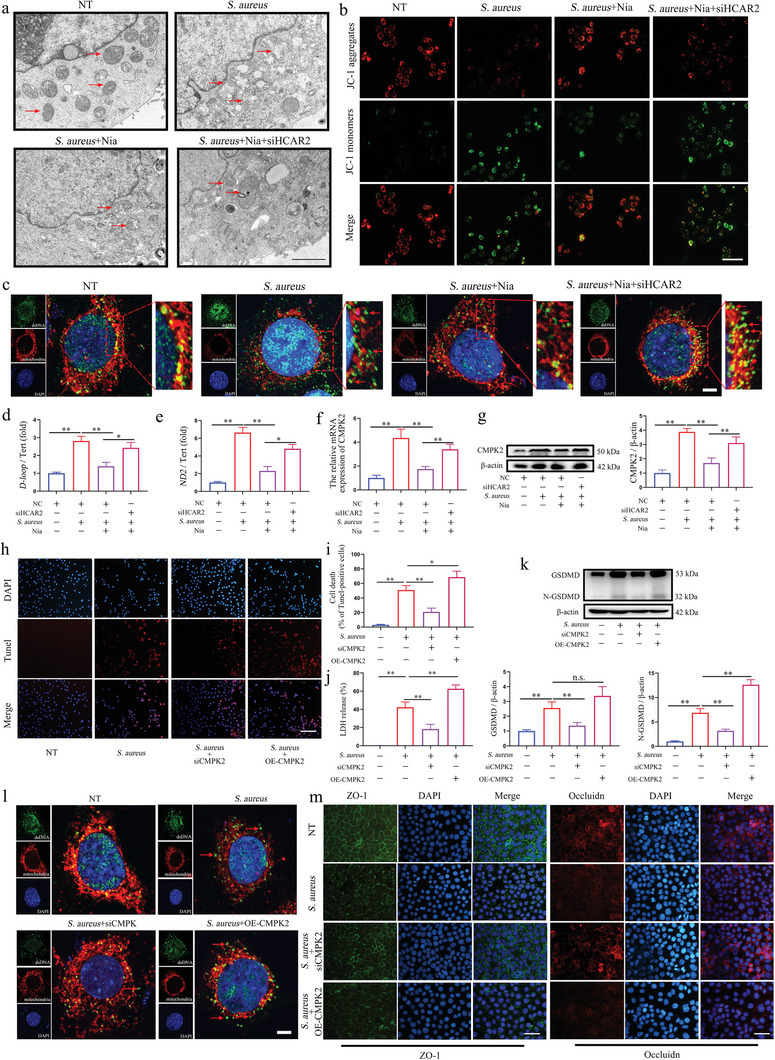
HCAR2 inhibits pyroptosis and tight junction damage in mMECs induced by *S. aureus* through downregulation of CMPK2 expression. a) Transmission electron microscopy was used to observe the mitochondrial morphology in mMECs, with red arrows indicating mitochondria and a scale bar of 2 µm. b) The mitochondrial membrane potential of mMECs was assessed using the JC‐1 membrane potential assay kit, with a scale bar of 100 µm. c) dsDNA antibodies and Mitotracker were used to label dsDNA and mitochondria, followed by imaging with a laser confocal microscope. Red arrows indicate co‐localization of dsDNA and mitochondria, with a scale bar of 2 µm. d, e) qPCR was performed to detect specific primers for mtDNA (D‐loop, ND2) and nDNA (Tert), and to determine the relative content of mtDNA. f) qRT‐PCR was used to measure mRNA levels of CMPK2 in mMECs. g) Western blot analysis was employed to detect CMPK2 protein expression in mMECs. h) TUNEL staining of mMECs, with red fluorescence representing TUNEL‐positive cells and a scale bar of 200 µm. i) Statistical analysis of TUNEL‐positive cells. j) LDH release was assessed using the LDH assay kit in mMECs. k) Western blot analysis was used to measure GSDMD protein expression in mMECs. l) dsDNA antibodies and Mitotracker were used to label dsDNA and mitochondria, followed by imaging with a laser confocal microscope. Red arrows indicate co‐localization of dsDNA and mitochondria, with a scale bar of 2 µm. m) Immunofluorescence of ZO‐1 and Occludin in mMECs, with a scale bar of 100 µm. Results are presented as mean ± SD, n = 3.

Given that in vivo experiments revealed exacerbated mitochondrial damage induced by *S. aureus* upon HCAR2 deletion, the mitochondrial status and function in mMECs were further evaluated. *S. aureus* induced the loss of mitochondrial cristae and rupture of the mitochondrial membrane in mMECs, whereas Nia mitigated this mitochondrial damage through HCAR2 activation (**Figure** **6**a). Furthermore, Nia restored the mitochondrial membrane potential reduced by *S. aureus* via HCAR2 (Figure 6b). Similarly, results from primary mMECs suggested that activating HCAR2 aids in repairing mitochondrial damage caused by *S. aureus* (Figure S4i, Supporting Information). Mitochondrial damage typically leads to the release of mtDNA, which functions as a DAMP to activate the NLRP3 inflammasome within the cell. To assess this, dsDNA and mitochondria were stained, revealing that Nia reduced the release of mitochondrial DNA (mtDNA) from mitochondria in *S. aureus*‐infected mMECs through HCAR2 activation (Figure 6c). Furthermore, disparities in the dsDNA content across groups led to the investigation of mtDNA replication. Subsequent experiments demonstrated that *S. aureus* stimulates enhanced mtDNA replication in mMECs, while Nia suppressed mtDNA replication via HCAR2 activation (Figure 6d,e). Previous research has shown that CMPK2 regulates mtDNA replication and contributes to inflammatory diseases by promoting this process.^[^ ^22^ ^]^ Based on in vivo sequencing results, CMPK2 levels were assessed in mMECs, revealing that HCAR2 activation effectively suppressed *S. aureus*‐induced CMPK2 overexpression in both mMECs and primary mMECs (Figure 6f,g; Figure S4j, Supporting Information).

Subsequently, the involvement of CMPK2 in *S. aureus*‐induced mMECs was investigated. The effects of siCMPK2 and OE‐CMPK2 on mMECs were evaluated, revealing that OE‐CMPK2 significantly increased CMPK2 expression in *S. aureus*‐stimulated mMECs, while siCMPK2 markedly reduced CMPK2 expression (Figure S5a–e, Supporting Information). Moreover, siCMPK2 and OE‐CMPK2 modulated pyroptosis in mMECs, with siCMPK2 mitigating and OE‐CMPK2 exacerbating pyroptosis induced by *S. aureus* (Figure 6h,i). LDH release assays corroborated these observations (Figure 6j). Additionally, OE‐CMPK2 promoted GSDMD activation, whereas siCMPK2 inhibited its activation (Figure 6k). In *S. aureus*‐stimulated mMECs, OE‐CMPK2, and siCMPK2 respectively enhanced and suppressed mtDNA release (Figure 6l). Both inhibition and overexpression of CMPK2 significantly decreased or increased mtDNA replication, respectively (Figure S5f,g, Supporting Information). Moreover, OE‐CMPK2 and siCMPK2 exacerbated and ameliorated TJ loss in mMECs induced by *S. aureus* (Figure 6m; Figure S5h,i, Supporting Information). Additionally, OE‐CMPK2 and siCMPK2 upregulated and downregulated the activation of the NLRP3 inflammasome in *S. aureus*‐stimulated mMECs, respectively (Figure S5j, Supporting Information). These results collectively demonstrate that activation of HCAR2 can inhibit CMPK2 over‐expression, thereby alleviating pyroptosis and tight junction damage caused by *S. aureus* in mMECs.

### 2.7 The Release of mMEC‐Derived Factors Following *S. aureus* Infection Modulates Macrophage Function and Activity via the cGAS/STING Pathway

**Figure 7:**
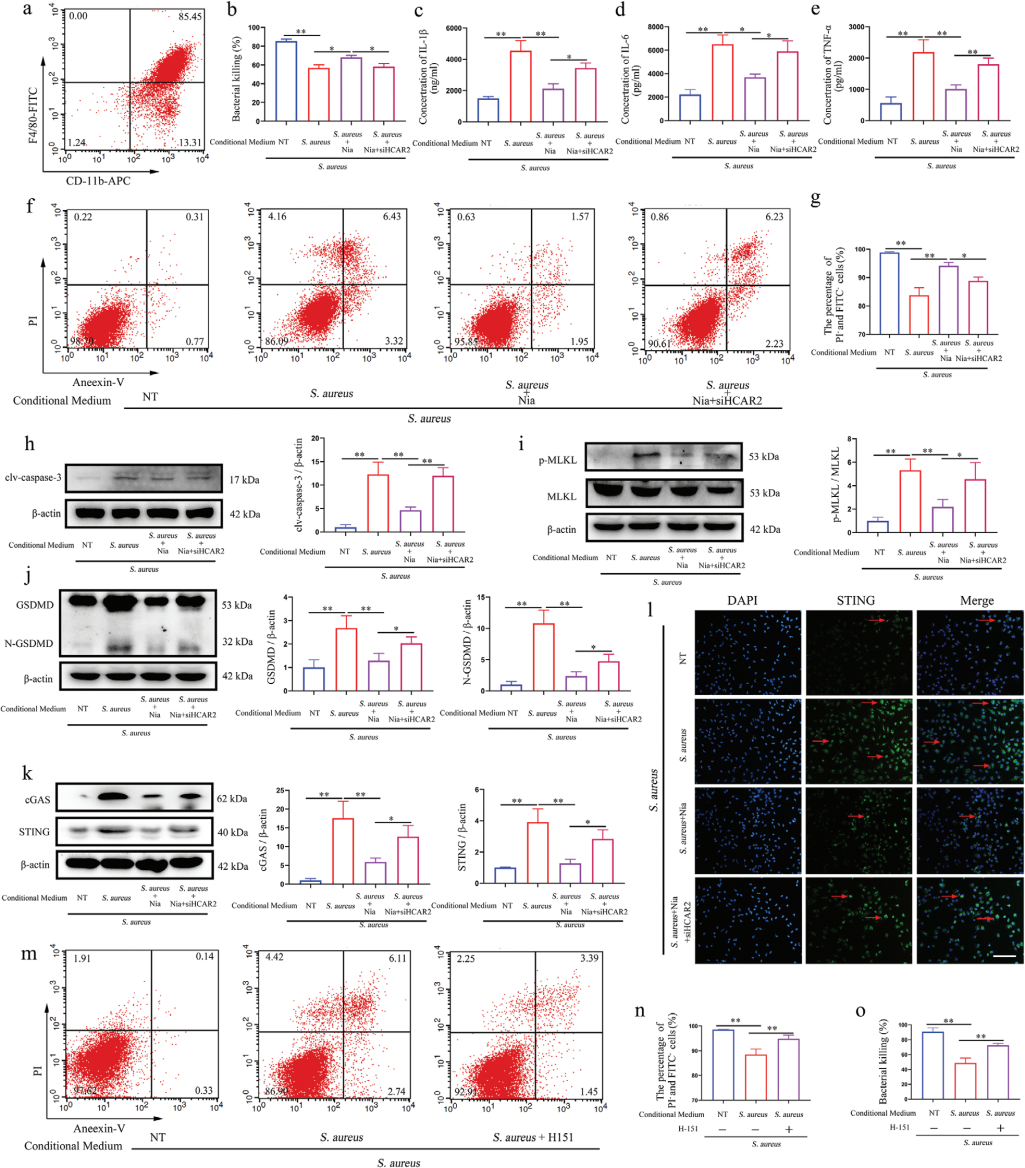
The release of mMEC‐derived factors following *S. aureus* infection modulates macrophage function and activity via the cGAS/STING pathway. a) The purity of primary peritoneal macrophages was assessed using F4/80 and CD11b labeling. After 6‐h incubation with supernatants from various mMEC groups, macrophages were subsequently infected with *S. aureus* for an additional 6 h. b) Macrophage bactericidal activity against *S. aureus* was measured by plate counting. c–e) ELISA was employed to quantify IL‐1β, IL‐6, and TNF‐α levels in the macrophage culture supernatant. f) Flow cytometry was performed to assess cell viability. g) The proportion of viable macrophages was determined in each group. h–j) Western blot analysis was used to detect the protein expression of cleaved caspase‐1, p‐MLKL, MLKL, and GSDMD. k) Western blot analysis was used to detect the protein expression of cGAS and STING. l) Immunofluorescence staining for STING in macrophages was visualized, with red arrows indicating STING localization (scale bar = 200 µm). Macrophages were pre‐incubated with 5 µm H‐151 for 1 h, followed by exposure to supernatants from the *S. aureus*‐treated mMEC group for 6 h, and subsequent infection with *S. aureus* for 6 h. m) Flow cytometry was again used to measure cell viability. n) The percentage of viable macrophages was quantified in each group. o) The bactericidal capacity of macrophages against *S. aureus* was assessed by plate counting. Results are presented as mean ± SD, n = 3.

Given that pyroptosis in mMECs precedes immune cell recruitment into the acinar lumen, it was hypothesized that pyroptosis in mMECs might also influence the functionality of native immune cells. Primary peritoneal macrophages and neutrophils were isolated from WT mice (**Figure** **7**a; Figure S6a, Supporting Information). To replicate the immune microenvironment in which macrophages and neutrophils migrate to a mammary gland infected with *S. aureus*, macrophages were exposed to the supernatant from mMEC cultures infected with *S. aureus*. The results revealed that the *S. aureus* group exhibited decreased bactericidal activity compared to the NT group, while the *S. aureus* + Nia group demonstrated enhanced bactericidal ability relative to the *S. aureus* group. However, the bactericidal ability of the *S. aureus* + Nia + siHCAR2 group was lower than that of the *S. aureus* + Nia group (Figure 7b). No significant difference was observed in the bactericidal ability of neutrophils among the groups (Figure S6b, Supporting Information). In contrast to the bactericidal ability, the release of pro‐inflammatory factors from macrophages showed an opposite trend (Figure 7c–e). Given that macrophage function is closely linked to cellular activity, the activity of each macrophage group was assessed. The results showed that cell activity in the *S. aureus* group was significantly lower than that in the NT group, whereas cell activity in the *S. aureus* + Nia group was higher than in the *S. aureus* group. The *S. aureus* + Nia + siHCAR2 group exhibited lower cell activity than the *S. aureus* + Nia group (Figure 7f,g). Interestingly, neutrophil activity displayed a similar trend to that of macrophages (Figure S6c,d, Supporting Information). Further analysis revealed that the expression of clv‐caspase‐1, p‐MLKL, and N‐GSDMD was significantly upregulated in the *S. aureus* group compared to the NT group, while expression was downregulated in the *S. aureus* + Nia group compared to the *S. aureus* group. In contrast, expression was up‐regulated in the *S. aureus* + Nia + siHCAR2 group compared to the *S. aureus* + Nia group (Figure 7h–j). These results suggest that co‐stimulation can trigger extensive PCDs in macrophages and that reducing mMEC pyroptosis via HCAR2 may alleviate PCDs in co‐stimulated macrophages.

Additionally, significant alterations were observed in the cGAS/STING signaling axis of macrophages and neutrophils following co‐stimulation, with changes correlating with the expression of PCD‐related proteins (Figure 7k,l; Figure S6e, Supporting Information). To further investigate this, the STING antagonist H‐151 was used to suppress STING signaling in macrophages and measured associated proteins. The results showed that H‐151 significantly inhibited the secretion of pro‐inflammatory factors in the *S. aureus* group (Figure S7a–c, Supporting Information). Macrophage activity and bactericidal ability in the *S. aureus* group were significantly improved by H‐151 (Figure 7m–o). Additionally, H‐151 reduced the expression of clv‐caspase‐1, p‐MLKL, and N‐GSDMD in the *S. aureus* group (Figure S7d,e, Supporting Information). These results underscore the role of STING in inducing widespread PCDs in macrophages through co‐stimulation.

### 2.8 Extracellular mtDNA is Critical for Activating the cGAS/STING Signaling Axis in Macrophages, with HCAR2 Deficiency Rendering them More Sensitive to mtDNA

**Figure 8:**
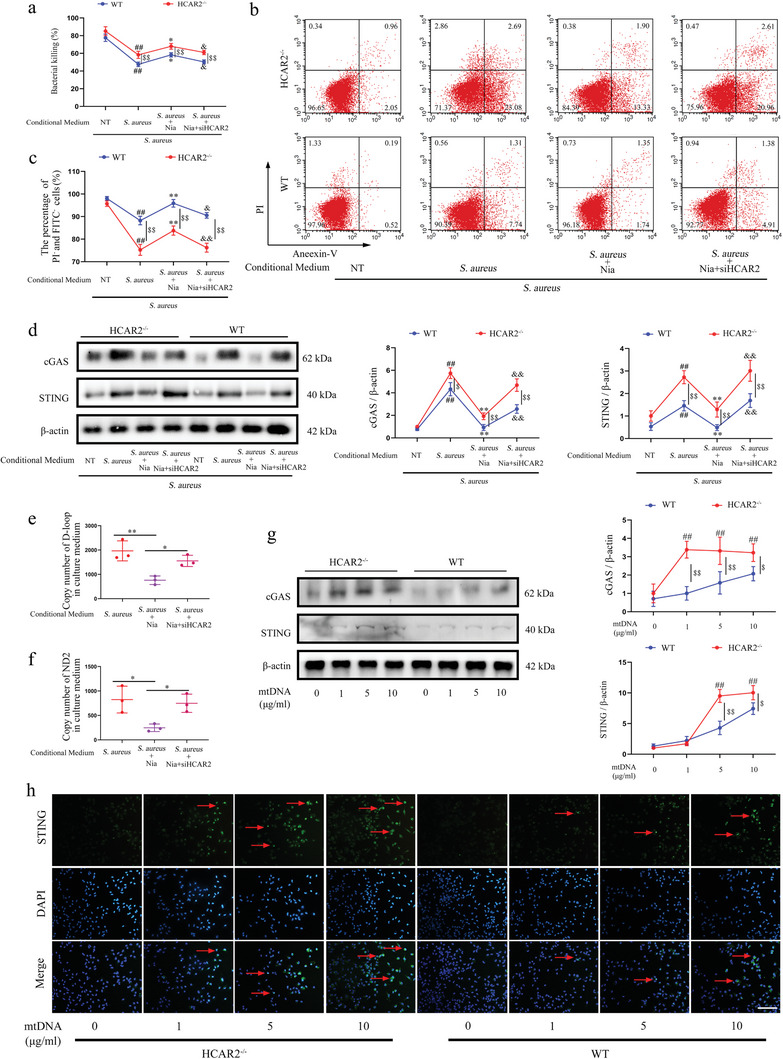
Extracellular mtDNA is critical for activating the cGAS/STING signaling axis in macrophages, with HCAR2 deficiency rendering them more sensitive to mtDNA. Macrophages were incubated with the supernatants from mMECs of various groups for 6 h, followed by infection with *S. aureus* for an additional 6 h. a) Macrophage bactericidal activity against *S. aureus* was assessed using a plate counting method. b) Cell viability was analyzed by flow cytometry. c) The percentage of normally active macrophages in each group was determined. d) Western blot analysis was performed to measure the protein expression of cGAS and STING. e, f) The copy number of D‐loop and ND2 in the supernatants of each group of mMEC cultures was quantified. Following incubation of macrophages with varying concentrations of mtDNA for 12 h, further experiments were carried out. g) Western blot was used to assess cGAS and STING protein levels. h) Immunofluorescence staining of STING in macrophages. The red arrow indicates STING, with a scale bar of 200 µm. Statistical significance is indicated as follows: ## *p* < 0.01 compared to the NT group; ^*^ and ^**^ *p* < 0.05 and *p* < 0.01, respectively, compared to the *S. aureus* group; & and && *p* < 0.05 and *p* < 0.01, respectively, compared to the *S. aureus* + Nia group; $ and $$ *p* < 0.05 and *p* < 0.01, respectively, compared to WT mice. Results are expressed as mean ± SD, n = 3.

Macrophage purity was assessed, revealing no difference between macrophages isolated from HCAR2^−/−^ and WT mice (Figure S8a, Supporting Information). Subsequently, macrophages were stimulated with *S. aureus* and the supernatants from mMEC cultures to evaluate their bactericidal activity (Figure S8b, Supporting Information). The results indicated that the bactericidal capacity of macrophages from HCAR2^−/−^ mice was comparable to that of WT mice, but the bactericidal ability in all other groups was significantly lower than that of WT mice, except the NT group (**Figure** **8**a). In addition, the secretion of pro‐inflammatory factors was significantly elevated in macrophages from all groups except the NT group, compared to the WT group (Figure S8c–e, Supporting Information). Assessment of cell viability revealed a marked reduction in macrophage activity in HCAR2^−/−^ mice in the *S. aureus*, *S. aureus* + Nia, and *S. aureus* + Nia + siHCAR2 groups, relative to WT mice (Figure 8b,c). cGAS/STING signaling changes in macrophages from HCAR2^−/−^ mice mirrored those in WT mice, but the expression levels of cGAS and STING were significantly lower in macrophages from HCAR2^−/−^ mice in all groups, except the NT group (Figure 8d). These results suggested that HCAR2‐deficient macrophages are more susceptible to the supernatant from *S. aureus*‐infected mMEC cultures.

Building on previous studies indicating that HCAR2 activation reduces CMPK2 expression in mMECs infected with *S. aureus*, leading to decreased mtDNA replication and pyroptosis, it was hypothesized that the variation in mtDNA released from mMECs could alter cGAS/STING signaling in macrophages. The data demonstrated that mtDNA copy numbers were lower in the *S. aureus*+Nia group compared to the *S. aureus* group, while the *S. aureus*+Nia+siHCAR2 group exhibited higher mtDNA levels than the *S. aureus* group (Figure 8e,f). This suggests that HCAR2 activation in *S. aureus*‐infected mMECs reduces mtDNA release. Further investigation, wherein mtDNA was extracted from mouse mammary tissue and used to stimulate macrophages at various concentrations, revealed that increasing mtDNA concentrations upregulated the protein expression of cGAS and STING in macrophages. Macrophages lacking HCAR2 were more responsive to mtDNA stimulation (Figure 8g). Immunofluorescence analysis of STING also confirmed these findings (Figure 8h). Moreover, mtDNA stimulation induced the secretion of pro‐inflammatory factors by macrophages, with HCAR2‐deficient macrophages producing higher levels of these factors (Figure S6f–h, Supporting Information). Additionally, in vivo, data demonstrated that the absence of HCAR2 enhances the activation of the cGAS/STING pathway and promotes widespread PCDs in the mammary glands of *S. aureus*‐infected mice (Figure S9, Supporting Information). Collectively, these results indicate that mtDNA released by *S. aureus*‐infected mMECs activates the cGAS/STING signaling axis in macrophages, impairing their function and activity.

### 2.9 CMPK2 Mediates the Alleviating Effect of Nia on *S. aureus* Infection in the Mammary Gland of Mice

**Figure 9:**
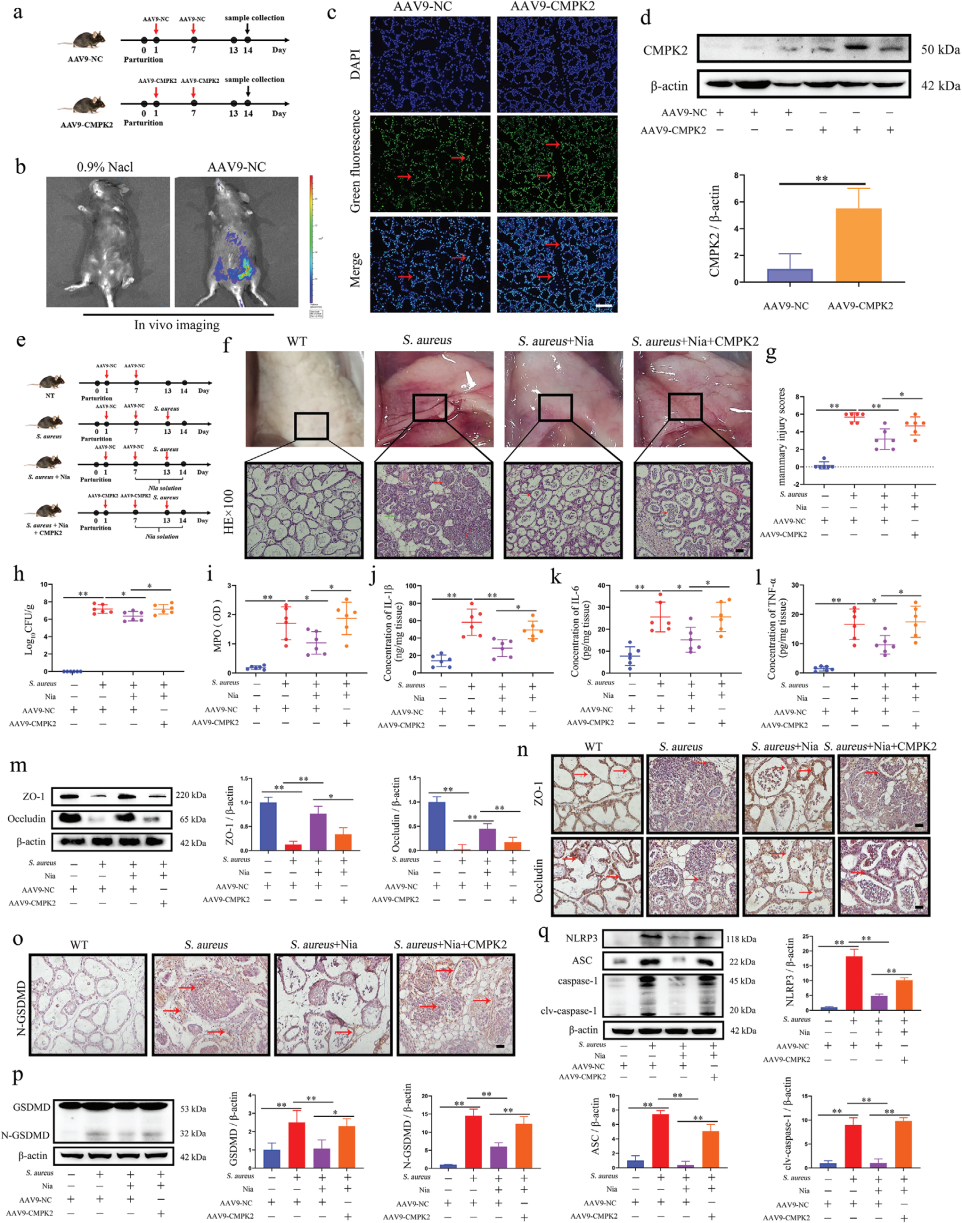
CMPK2 mediates the alleviating effect of Nia on S. aureus infection in the mammary gland of mice a) Flowchart illustrating intra‐mammary inoculation with adenovirus. b) In vivo imaging of the mammary gland following injection of 0.9% NaCl or adenovirus. c) Intramammary fluorescence detection of AAV9‐NC and AAV9‐CMPK2 after inoculation into the mammary gland, with green fluorescence indicating adenovirus and blue fluorescence representing the nucleus; scale bar represents 50 µm. d) Western blot analysis of CMPK2 protein expression in mammary tissue. e) Flowchart of the animal experimental procedure. f) Representative images of mouse mammary gland and H&E staining; red arrows indicate inflammatory cell infiltration, green arrows indicate thickening of the acinar wall; scale bar, 100 µm. g) Mammary injury score. h) Bacterial load in mammary tissue. i) MPO content in mammary tissue. j–l) ELISA analysis of IL‐1β, IL‐6, and TNF‐α protein levels in mammary tissue. m) Western blot analysis of ZO‐1 and Occludin protein expression in mammary tissue. n) IHC for ZO‐1 and Occludin in mammary tissue; scale bar, 50 µm. o) IHC for N‐GSDMD in mammary tissue; scale bar, 50 µm. p) Western blot analysis of GSDMD protein expression in mammary tissue. q) Western blot analysis of NLRP3, ASC, and caspase‐1 protein expression in mammary tissue. Results are presented as mean ± SD, n = 6.

Based on the previous findings, alterations in CMPK2 expression within mammary tissue were further investigated, revealing that Nia significantly inhibited the protein levels of CMPK2, cGAS/STING, and key executive proteins involved in PCDs in *S. aureus*‐induced mastitis in mice (Figure S10, Supporting Information). To determine whether HCAR2 mediates the resolution of mastitis caused by *S. aureus* in the mammary gland via CMPK2, CMPK2 was reintroduced into the mammary gland using adenovirus vectors, and the effects on mastitis amelioration by the HCAR2 agonist were assessed. Upon injection of two adenoviruses, AAV‐NC and AAV‐CMPK2, into the mammary ducts of mice, in vivo imaging demonstrated robust fluorescence signals in the mammary gland of the AAV9‐treated group, compared to the negative control (0.9% NaCl), confirming successful colonization of the mammary glands by the adenovirus vector (**Figure** **9**a,b). Further analysis showed stable expression of both AAV9‐NC and AAV9‐CMPK2 in mammary tissues, with AAV9‐CMPK2 significantly increasing CMPK2 protein expression in the gland (Figure 9c,d). Next, Nia was used to activate HCAR2, and a mastitis model was established with *S. aureus* (Figure 9e). The findings indicated that while Nia alleviated the symptoms of *S. aureus*‐induced mastitis and reduced the bacterial load in the mammary gland, this improvement was significantly reversed upon CMPK2 overexpression (Figure 9f–h). Additionally, MPO and pro‐inflammatory factor assays showed that CMPK2 overexpression in the mammary glands of *S. aureus*‐infected mice diminished the inhibitory effect of Nia on MPO and pro‐inflammatory factors (Figure 9i–l). Further analysis of the blood‐milk barrier revealed that CMPK2 weakened Nia's protective effects on this barrier (Figure 9m,n). Lastly, assessments of cell pyroptosis in mammary tissue showed that CMPK2 compromised Nia's ability to reduce cell pyroptosis and weakened Nia's inhibitory effect on NLRP3 inflammasome activation (Figure 9o–q). These results collectively demonstrate that CMPK2 mediates the alleviating effects of Nia on *S. aureus* infection in the mammary gland.

## 3 Discussion

The innate immune system, composed of epithelial cells and macrophages, plays a critical role in the early detection and elimination of pathogens that invade mammary tissue. However, epithelial cell death or an exaggerated immune response from macrophages can exacerbate pathological progression during infection.^[^ ^23^, ^24^ ^]^ This study demonstrates that activation of HCAR2 in *S. aureus*‐infected mMECs leads to a reduction in CMPK2/mtDNA expression, which in turn inhibits NLRP3 inflammasome activation and mitigates cell pyroptosis. The attenuation of pyroptosis in mMECs reduces the release of mtDNA into the extracellular space, thereby alleviating the suppression of macrophage activity induced by *S. aureus* via the cGAS/STING‐dependent pathway. These findings offer new insights into the pathogenesis of *S. aureus* mastitis and highlight potential therapeutic targets for managing *S. aureus*‐associated infections.

GPCRs are a crucial class of cell surface receptors with significant regulatory roles in bacterial infections across various organs.^[^ ^25^ ^]^ In the respiratory tract, the activation of GPCRs, particularly OXGR1 expressed on respiratory epithelial cells, is essential for maintaining epithelial cell function and regulating the innate immune response during bacterial infection.^[^ ^26^ ^]^ Similarly, research by Naina Gour et al. has shown that Mrgpra1, a GPCR located on the surface of neutrophils, plays a critical role in modulating excessive neutrophil responses during acute lung infections by regulating neutrophil polarization.^[^ ^27^ ^]^ To investigate GPCRs with immune regulatory functions during *S. aureus* infection, transcriptomic analysis revealed significant upregulation of HCAR2 expression in the mammary gland during infection. Previous studies by Guo et al. have demonstrated that the absence of HCAR2 exacerbates sepsis progression, while its immunoregulatory function has been confirmed in inflammatory conditions, such as enteritis and neuroinflammation.^[^ ^20^, ^28^, ^29^ ^]^ Our results indicate that HCAR2 deficiency exacerbates the inflammatory response, compromises barrier integrity, and promotes increased bacterial colonization within the mammary gland. In contrast, administration of HCAR2 agonists alleviated these pathological changes.

HCAR2 is highly expressed not only in macrophages and neutrophils but also in mMECs, where it plays a critical role in regulating milk fat composition.^[^ ^19^, ^30^ ^]^ As the predominant cell type in the breast, mMECs are integral to both milk secretion and the formation of the blood‐milk barrier, contributing significantly to immune regulation and the maintenance of the mammary gland's internal environment.^[^ ^31^ ^]^ Disruption or dysfunction of these cells compromises the blood‐milk barrier, alters the mammary gland's internal environment, and adversely affects immune cell function.^[^ ^11^, ^32^ ^]^ Numerous studies have confirmed that the death of mMECs during bacterial infections accelerates the destruction of the blood‐milk barrier, leading to tissue damage.^[^ ^33^, ^34^ ^]^ Findings from both in vivo and in vitro experiments in this study indicate that HCAR2 activation can restore the integrity of the blood‐milk barrier damaged by *S. aureus*. Previous research has highlighted the role of pyroptosis in combating immune evasion by intracellular bacteria^[^ ^35^ ^]^; however, excessive pyroptosis can result in irreversible tissue damage and systemic inflammatory consequences.^[^ ^36^ ^]^ Recent studies by Chao Wei have emphasized the importance of endothelial cell pyroptosis in the disruption of the inflammatory blood‐brain barrier,^[^ ^37^ ^]^ while Xiaozhou Wang et al. demonstrated that *S. aureus* triggers pyroptosis in mMECs.^[^ ^12^ ^]^ This study shows that HCAR2 activation significantly mitigates *S. aureus*‐induced pyroptosis in mMECs.

The NLRP3 inflammasome can be activated by intracellular *S. aureus*, leading to the assembly of a protein complex comprising NLRP3, ASC, and caspase‐1. This complex subsequently activates caspase‐1 to cleave caspase‐1 and GSDMD, producing the N‐terminal fragment of GSDMD, ultimately resulting in pyroptosis.^[^ ^38^ ^]^ In this study, it was found that in mice with *S. aureus*‐induced mammary gland infections, the deletion of HCAR2 enhances the activation of the NLRP3 inflammasome, while HCAR2 activation mitigates NLRP3 activation triggered by *S. aureus*. Mitochondrial damage plays a pivotal role in the induction of pyroptosis and inflammation. Mitochondrial components and metabolic byproducts, released as DAMPs into the extracellular space, further propagate inflammation in infectious diseases.^[^ ^39^ ^]^ Upon mitochondrial damage, mtDNA undergoes oxidation and translocates to the cytoplasm, where it directly interacts with NLRP3 to activate the inflammasome.^[^ ^40^ ^]^ Suyuan Liu et al. demonstrated that inhibiting mitochondrial DNA synthesis effectively modulates inflammatory responses mediated by the NLRP3 inflammasome.^[^ ^41^ ^]^ Similarly, Xing Gao et al. showed that *S. aureus* induces mitochondrial damage in mMECs, leading to elevated mitochondrial reactive oxygen species (mtROS) levels and electron transport chain (ETC) dysfunction.^[^ ^42^ ^]^ This study further demonstrates that HCAR2 activation, both in vivo and in vitro, alleviates *S. aureus*‐induced mitochondrial damage and significantly reduces the upregulation of mtDNA duplication in *S. aureus*‐stimulated mMECs.

CMPK2, a mitochondrial nucleotide monophosphate kinase essential for deoxyribonucleoside triphosphate (dNTP) biosynthesis,^[^ ^43^, ^44^ ^]^ has been shown to regulate mtDNA synthesis and inhibit NLRP3 inflammasome activation.^[^ ^45^ ^]^ Previous studies have indicated that targeting CMPK2 to reduce mtDNA synthesis can effectively suppress NLRP3 inflammasome activation, thus mitigating pneumonia and sepsis infections.^[^ ^22^ ^]^ In this study, CMPK2 was identified as a downstream target of HCAR2, and modulation of CMPK2 expression in mMECs was found to influence both NLRP3 inflammasome activation and pyroptosis in these cells. Meanwhile, CMPK2 mediated the alleviating effect of Nia on *S. aureus* infection in the mammary gland of mice. In mMECs stimulated with *S. aureus*, CMPK2 expression was closely associated with mitochondrial stability, possibly linked to CMPK2's role in regulating GSDMD activation. Rui Miao et al. demonstrated that N‐GSDMD interacts with the outer mitochondrial membrane, leading to mitochondrial damage within cells.^[^ ^37^ ^]^ Nevertheless, the precise regulatory mechanism by which HCAR2 influences CMPK2 remains to be elucidated. This will also serve as the subsequent experimental direction for this study.

This study demonstrates that both WT and HCAR2^−/−^ mice exhibit pyroptosis in the mammary glands following *S. aureus* infection, with the latter group displaying a more pronounced degree of pyroptosis. Additionally, the bacterial load in the mammary glands of HCAR2^−/−^ mice is significantly elevated, suggesting that HCAR2 may regulate epithelial pyroptosis and influence the bactericidal capacity of immune cells. Previous research has indicated that the interaction between alveolar epithelial cells and macrophages can impair macrophage phagocytic function in chronic obstructive pulmonary disease.^[^ ^46^ ^]^ Similarly, hepatocyte pyroptosis exacerbates acute liver injury by enhancing macrophage‐mediated inflammatory responses.^[^ ^47^ ^]^ A significant correlation between mMEC apoptosis and macrophage activity has also been reported, particularly highlighting M2 macrophages as key contributors to epithelial cell death during postpartum breast tissue remodeling.^[^ ^48^ ^]^ However, the role of the interaction between mammary epithelial cell death and macrophages in breast immunity remains largely unexplored. While previous studies suggest that pyroptosis in epithelial or endothelial cells can enhance macrophage bactericidal functions by preventing bacterial immune evasion,^[^ ^49^ ^]^ our findings confirm that pyroptosis of mMECs exacerbates inflammatory responses in *S. aureus*‐infected macrophages, reducing their activity and bactericidal capacity. Furthermore, this study shows that HCAR2 modulates pyroptosis in mMECs to alleviate the reduction in macrophage activity induced by *S. aureus*. Research by Wang Y and Chen X et al. has shown that macrophage death leads to an increased release of pro‐inflammatory mediators, impairing their bactericidal capacity, whereas promoting macrophage survival can reduce inflammation and restore cellular function.^[^ ^50^, ^51^ ^]^ Therefore, the diminished bactericidal capacity of macrophages in HCAR2^−/−^ mice is likely linked to reduced macrophage activity, which may also explain the observed increase in both bacterial load and inflammatory responses in the mammary glands of these mice.

Due to its ability to be activated by both endogenous and exogenous DNA, combined with the predominant expression of STING in immune cells, the cGAS‐STING signaling pathway plays a critical role in mediating inflammatory responses and bacterial infections.^[^ ^52^ ^]^ Furthermore, the involvement of cGAS‐STING in modulating various forms of PCDs)in macrophages has been well‐documented.^[^ ^53^, ^54^ ^]^ This study demonstrates that the reduction in macrophage activity caused by mMEC pyroptosis is regulated through the cGAS‐STING signaling pathway. Previous studies have shown that hepatocyte pyroptosis promotes the release of mtDNA into the extracellular space, thereby activating the cGAS‐STING pathway in macrophages.^[^ ^47^ ^]^ The present study demonstrated that pyroptosis in mMECs facilitates the extracellular release of mtDNA, which subsequently activates the cGAS‐STING signaling pathway in macrophages. Both in vivo and in vitro analyses revealed that HCAR2^−/−^ macrophages exhibit an enhanced capacity to activate the cGAS‐STING pathway. However, the exact mechanisms driving the increased susceptibility of HCAR2^−/−^ macrophages to mtDNA remain to be clarified.

**Figure 10:**
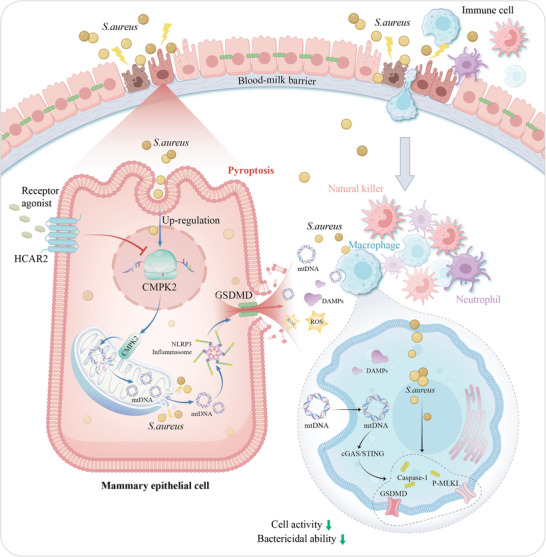
HCAR2 modulates the crosstalk between mammary epithelial cells and macrophages to mitigate *S. aureus* infection in the mouse mammary gland.

In conclusion, our findings demonstrate that the activation of HCAR2 in *S. aureus*‐infected mMECs effectively reduces CMPK2/mtDNA expression, thereby inhibiting NLRP3 inflammasome activation and mitigating pyroptosis. The attenuation of pyroptosis in mMECs leads to a decreased release of mtDNA, which subsequently inhibits the activation of the cGAS/STING signaling pathway in macrophages, alleviating the reduced macrophage activity induced by *S. aureus* in a cGAS/STING pathway‐dependent manner (**Figure** **10**). These results not only elucidate the role of HCAR2 in enhancing resistance to *S. aureus* infection but also provide insight into the underlying mechanisms, establishing HCAR2 as a potential therapeutic target for *S. aureus*‐induced mastitis.

## 4 Experimental Section

### Experimental Animals and Design

In this experiment, 9‐week‐old wild type (WT) C57BL/6 were obtained from Liaoning Changsheng Biotechnology Co. and HCAR2^−/−^mice were generous gift from Dr. Martin Sager (Zentrale Einrichtung für Tierforschung und Tierschutzaufgaben der Heinrich‐Heine Universität Düsseldorf, Germany). One male and one female mouse were placed in the same cage and allowed to eat and drink freely. After the mice became pregnant, the pregnant mice were transferred to a new cage for the experiment. All animal experiments were carried out in strict adherence to the guidelines set forth by the Animal Welfare Protection and Utilization Committee of Jilin University (SY202409026).

The mouse experiments were divided into four parts. 1) Within 12–14 days postpartum, WT mice were randomly assigned to two groups: the NT group and the *S. aureus* group, with 6 mice per group. All mice were separated from their pups 2 h before model induction. The *S. aureus* group was used to establish the *S. aureus*‐induced mastitis model. 2) Within 12–14 days postpartum, WT mice were randomly divided into two groups: the WT group and the WT+*S. aureus* group, with 6 mice per group. HCAR2^−/−^ mice were also divided into two groups: the HCAR2^−/−^ group and the HCAR2^−/−^+*S. aureus* group, with 6 mice per group. The WT+*S. aureus* and HCAR2^−/−^+*S. aureus* groups were used to establish the *S. aureus*‐induced mastitis model. 3) Within 5–7 days postpartum, WT mice were randomly assigned to three groups: the NT group, the *S. aureus* group, and the Nia + *S. aureus* group, with 6 mice per group. Mice in the Nia + *S. aureus* group were administered a 50 mm nicotinic acid (Nia) solution dissolved in drinking water for 7 days before model induction. Both the *S. aureus* and Nia + *S. aureus* groups were used to establish the *S. aureus*‐induced mastitis model. 4) Within 1 day postpartum, WT mice were randomly assigned to four groups: the NT group, the *S. aureus* group, the Nia + *S. aureus* group, and the Nia + *S. aureus* + CMPK2 group, with 6 mice per group. All mice received a 50 µL adenovirus injection into the subcutaneous mammary tissue on the 1st and 7th days postpartum, with a viral titer of 1 × 10^12^ V.g mL^−1^. AAV9‐NC was injected into the NT, *S. aureus*, and Nia + *S. aureus* groups, while AAV9‐CMPK2 was injected into the Nia + *S. aureus* + CMPK2 group. Seven days before model induction, the Nia + *S. aureus* and Nia + *S. aureus* + CMPK2 groups were administered a 50 mm nicotinic acid solution in their drinking water. The *S. aureus*, Nia + *S. aureus*, and Nia + *S. aureus* + CMPK2 groups were used to establish the *S. aureus*‐induced mastitis model. D‐luciferin sodium salt was employed as a luminescent substrate to observe adenovirus replenishment in the mammary gland tissue using an in vivo small animal 3D visible light imaging system (IVIS Spectrum). The Living Image 4.3 software was used for post‐imaging analysis.

### *S. aureus*‐Induced Mastitis Mice Model Construction

Mice were anesthetized by intraperitoneal injection of 45 mg kg^−1^ pentobarbital sodium, and once anesthesia was achieved, they were placed supine and secured. The region surrounding the fourth pair of mammary glands was disinfected with 75% ethanol. Using fine tweezers, the nipple was raised, and ≈2 mm of the tip was excised under an anatomical microscope. A 100 µL micropipette was employed to inject 50 µL of a 1 × 10^8^ CFU mL^−1^ *S. aureus* suspension into the nipple (the *S. aureus* suspension was prepared in PBS at the appropriate concentration).^[^ ^55^ ^]^ In control groups, 50 µL of sterile PBS was injected into the mammary gland. After 24 h, all mice were humanely euthanized. The fourth pair of mammary glands were then exposed, carefully observed, and photographed. Mammary tissue samples were collected for subsequent analysis.

### Transcriptome Sequencing

Following the collection of mouse mammary tissues, sequencing and data analysis were conducted by Lianchuan Biomedical Technology Co (Hangzhou, China). In summary, after collecting mouse mammary tissue, RNA was extracted and purified, and its concentration and purity were assessed using the NanoDrop ND‐1000. The RNA was then reverse‐transcribed to generate complementary DNA (cDNA). The RNA‐DNA hybrid was subsequently converted into double‐stranded DNA, with the ends capped using specialized reagents. The double‐stranded DNA was digested with UDG enzyme, and a library was constructed using PCR amplification, targeting fragment sizes of 300 bp ±50 bp. Sequencing was performed using the Illumina NovaSeq 6000 (LC Bio‐Technology Co., Ltd., Hangzhou, China), following standard protocols. Sequencing data were aligned to the Homo sapiens GRCh38 genome using HISAT2 (https://ccb.jhu.edu/software/hisat2). Differential gene expression analysis was conducted with the R package edgeR (https://bioconductor.org/packages/release/bioc/html/edgeR.html). GO and KEGG pathway enrichment analyses were performed using DAVID software (https://david.ncifcrf.gov/).

### Hematoxylin−Eosin (H&E) Staining

The tissue sections were deparaffinized using xylene, followed by rehydration with a series of ethanol solutions. The sections were subsequently stained with hematoxylin and eosin, dehydrated in ethanol, cleared in xylene, and ultimately mounted with neutral resin. The criteria for the mammary injury score were established based on a previous study.^[^ ^56^ ^]^

### Immunohistochemical (IHC)

After the sections of mammary tissue were dewaxed and hydrated, immerse the sections in citrate buffer for antigen retrieval. Subsequently, the sections were rinsed with PBS and a peroxidase blocker was applied. The sections were inoculated with 5% donkey serum to block, followed by incubation with primary antibody diluted in 5% donkey serum at 4 °C overnight. Then the immunohistochemistry kit (Biological Technology, Wuhan, China) was used for immunohistochemical staining, and finally, neutral resin was used to mount the slides.

### Immunofluorescence (IF)

After the sections of mammary tissue were dewaxed and hydrated, the antigen was repaired using citrate buffer, and the endogenous peroxidase was blocked using a paraffin embedding compound. After the cells were inoculated and treated on the cell crawl, they were fixed with immunostaining fixative. The subsequent procedures were conducted in a manner consistent with the cellular protocol, involving surface blocking using 5% donkey serum, subsequent incubation with the primary antibody, and application of a fluorescent secondary antibody. Finally, the coverslips containing DAPI were used for staining and mounting, and fluorescence microscopy or laser scanning microscopy was used to observe the results.

### Blood‐Milk Barrier Integrity Assessment via FITC‐Albumin

FITC‐albumin (Sigma–Aldrich, USA) was dissolved in PBS, and freshly collected mammary tissue from each group was immersed in the FITC‐albumin solution for 45 min. Tissue samples were then processed for frozen sectioning, and the green fluorescence within the acini was visualized under a fluorescence microscope. Detailed experimental procedures could be found in a previous publication.^[^ ^57^ ^]^

### Detection of Myeloperoxidase (MPO) Activity

A little mammary tissue was taken and weighed, then the solution of N‐2‐Hydroxyethylpiperazine‐N‐2‐Ethane sulfonic acid 5000 times the weight was added to the tissue with magnetic beads, and then ground with automatic grinder for 10 min. After centrifugation, the supernatant was absorbed as a sample of elisa. Then, an equal volume of 5% cetyltrimethylammonium chloride solution was added to the HEPEs solution and continued grinding for 10 min. The supernatant was collected as the sample of MPO. Sequentially 75 µL of the sample was added to each well of the 96‐well plate, followed by the addition of 75 µL of MPO substrate coloring solution. Terminate the color reaction after 3 min by adding 2 m H_2_SO_4_ solution.

### Enzyme‐Linked Immunosorbent Assay (ELISA)

The instructions of the Elisa kit (Biolegend, San Diego, CA, USA) were followed for detecting IL‐1β, IL‐6, and TNF‐α in the mammary tissue of mice or the supernatant of macrophages.

### Western Blot

The tissue or cells were resuspended in NP40 lysate and transfer 2 mL into EP tubes. Following lysis, centrifuge to collect the supernatant as the protein sample, then utilize the BCA reagent kit (Thermo Scientific, USA) for protein concentration determination. Subsequently, aliquot the samples into separate tubes based on their protein concentration. Then, the 12% sodium dodecyl sulfate‐polyacrylamide gel electrophoresis (SDS‐PAGE) was performed. After transferring the membrane using the PVDF membrane, incubate the PVDF membrane with the primary antibody and secondary antibody. Finally, the electrochemiluminescence detection kit (Biyuntian, Wuhan, China) was used for the visualization of the protein bands by using an autoradiography apparatus. The information on antibodies used in this study is listed in Table S1 (Supporting Information).

### Quantitative Real‐Time Polymerase Chain Reaction (q‐RTPCR)

The total mRNA of tissue or cell was extracted by using Trizol (Thermo Fisher Scientific, Shanghai, China). Subsequently, RNA reverse transcription was conducted utilizing the cDNA First‐Strand Synthesis Kit (ABclonal, Wuhan, China). Finally, the Quantitect SYBR Green RT‐PCR kit (Roche, Shanghai, China) was used for q‐RTPCR. The primer sequence is listed in Table S2 (Supporting Information).

### *S. aureus* Infection of Cells

The mouse mammary epithelial cell line, EpH4‐Ev cells, was cultured in DMEM medium (Gibco, Grand Island, NY, USA) supplemented with 10% fetal bovine serum (Hyclone, USA). Cells in Petri dishes were counted, and *S. aureus* was added at a multiplicity of infection (MOI) of 10. After a 2‐h incubation with the bacteria, the medium was discarded, and the cells were washed twice with PBS. Gentamicin was then added to the medium at a final concentration of 100 µg mL^−1^ for 1 h, followed by two PBS washes. Fresh medium was added, and cells were cultured for an additional 4 or 6 h before the medium or cells were collected for further analysis.

### Measurement of Total mtDNA

Cells were collected, and total DNA was extracted using the Cell Fast DNA Extraction Kit (ABclonal, Wuhan, China) according to the manufacturer's instructions. Total mtDNA was quantified by qPCR using specific primers targeting the D‐loop region of mitochondria and other uninsured mtDNA regions. Nuclear DNA encoding Tert served as a normalization control. Detailed procedures could be found in previous studies.^[^ ^22^ ^]^ The primer sequences are provided in Table S2 (Supporting Information).

### Cell Viability

One‐step TUNEL In Situ Apoptosis Kit (Green, FITC) (Elabscience, Wuhan, China) was used to detect dead cells in the mammary gland. One Step TUNEL Apoptosis Assay Kit (Biyuntian, Wuhan, China) was used to detect dead cells of mMECs. Lactate dehydrogenase (LDH) Cytotoxicity Assay Kit (Biyuntian, Wuhan, China) was used to detect LDH released by cells. The Annexin V‐FITC Apoptosis Detection Kit (Biyuntian, China) was employed for assessing cell viability. All experiments were carried out in accordance with the instructions provided by the kit following cell treatment.

### RNA Interference and Overexpression

RNA interference and overexpression were performed using Lipofectamine 2000 (Thermo Fisher Scientific Co., Shanghai, China). Briefly, siRNA or overexpression plasmid and Lipofectamine 2000 were diluted in Opti‐MEM and then mixed for 20 min. The mixture was added to the cell culture dish, the medium was replaced after 6 h, and the cells were cultured for an additional 24 h. siHCAR2, siCMPK2, and OE‐CMPK2 were synthesized by Shanghai Genechem Co. Ltd. The primer sequences are listed in Table S3 (Supporting Information).

### Mitochondrial Probe Staining

Mitotracker Red CMXRos (Beyotime, Wuhan, China) was diluted in DMSO to a final concentration of 200 µm. The Mitotracker Red CMXRos solution was added to the cell culture medium at a 1:1000 dilution, and cells were incubated for 20 min. Cells were then fixed with an immunofixative solution, followed by immunofluorescence staining and subsequent procedures.

### Mitochondrial Membrane Potential Detection

The mitochondrial membrane potential assay kit with JC‐1 (Biyuntian, Wuhan, China) was used to detect changes in cell intima potential. Briefly, after the cells were treated, the cells medium was discarded and the JC‐1 dyeing solution was mixed with the new DMEM medium at a ratio of 1:1, and the cells were incubated with the mixture for 20 min. Then the cell medium was discarded, the cells were washed with JC‐1 staining buffer twice, and the results were observed by fluorescence microscopy.

### Extraction of Primary Mammary Epithelial Cells

Lactating mice were euthanized and their mammary glands were immersed in 75% ethanol for 30 s. The glands were dissected, and the lymph nodes within the tissue were removed. Mammary tissue was placed in a D‐Hanks solution containing antibiotics and washed at least 5 times. After removing the excess solution, the tissue was minced into small pieces. The tissue was transferred to 15 mL centrifuge tubes and incubated with trypsin at 37 °C for 2 h with shaking. After digestion, the samples were centrifuged at 1500 rpm for 10 min, the supernatant was discarded, and the pellet was resuspended in a 5 mL DMEM culture medium. Centrifugation at 1500 rpm for 5 min followed, and the supernatant was discarded. To eliminate red blood cells, 2 mL of Red Blood Cell Lysis Buffer was added at 37 °C and incubated for 3 min. Following a final centrifugation at 1500 rpm for 5 min, the supernatant was discarded. The cells were resuspended in a DMEM culture medium, filtered through a 40 µm cell strainer, and cultured for identification.

### Extraction of Primary Peritoneal Macrophages

Thioglycolate broth (3 mL) was injected into the peritoneal cavity of mice 3 days and 1 day before the experiment. Following euthanasia and immersion in 75% ethanol for 3 min, the mice were placed on a sterile operating table and 3 mL of complete 1640 culture medium was injected into the peritoneal cavity. The peritoneal cavity was gently massaged, and the peritoneal fluid was aspirated. The collected fluid was centrifuged at 1000 rpm for 5 min, and the supernatant was discarded. The pellet was resuspended in red blood cell lysate and mixed thoroughly. After another centrifugation at 1000 rpm for 5 min, the supernatant was discarded, and the pellet was resuspended in a DMEM medium. The suspension was centrifuged at 1000 rpm for 3 min, the supernatant was discarded, and the pellet was resuspended in a complete medium. After cell counting, the cell density was adjusted to 2 × 10^6^ cells mL^−1^, and the cells were seeded into 6‐well plates or 3 cm^2^ culture dishes according to experimental requirements.

### Extraction of Primary Peritoneal Neutrophils

Mice received two injections of 1 mL thioglycolate broth into the peritoneal cavity, with a 12‐h interval between injections. 3 h after the second injection, the mice were euthanized, and 3 mL of 1640 culture medium was infused into the peritoneal cavity. The peritoneal cavity was gently massaged for 2 min, and the peritoneal wash fluid was aspirated into 15 mL centrifuge tubes, repeating this step twice. The tubes were centrifuged at 1000 rpm for 10 min, and the supernatant was discarded. The collected cells were resuspended in a 1640 culture medium and red blood cell lysate. After adding granulocyte separation liquid, the mixture was centrifuged at 1500 rpm for 20 min. The lower white cell layer was collected into another 15 mL centrifuge tube and resuspended in a 5 mL 1640 culture medium. The suspension was centrifuged at 500 rpm for 5 min, the supernatant was discarded, and the step was repeated. The collected cells were analyzed for purity using fluorescently conjugated antibodies, including Ly6G‐FITC and CD11b‐APC.

### Extraction of mtDNA from Mammary Tissue

The Mitochondrial DNA Isolation Kit (Abcam, USA) was used to isolate mtDNA from mammary tissue. Briefly, mammary tissue was added with an appropriate amount of cytosol extraction buffer. After grinding, the mammary tissue was incubated for 10 min, centrifuged at 700 g for 10 min, supernatant was taken, and centrifuged at 10000 g for 30 min. After discarding the supernatant, the precipitation was suspended in the cytoplasmic extraction buffer briefly and centrifuged for 30 min. The supernatant was discarded and added to the mitochondrial cleavage buffer, followed by enzyme mix and ethanol for incubation for 10 min. Finally, the supernatant was discarded after centrifugation to obtain mtDNA precipitation.

### The Bactericidal Ability of Macrophages

Following 6 h incubation of macrophages with various culture supernatants of mMECs, the culture medium was replaced, and *S.aureus* (MOI = 5) was introduced to infect the macrophages for 2 h. After washing the cells with the culture medium twice, 100 µg mL^−1^ of gentamicin should be added to the culture medium, followed by incubation for 1 h. After washing the cells with culture medium twice, 0.25% Triton‐100 sterile water was added to the culture hole for cell lysis. The sterile water was used to be coated on the LB solid culture plate, and the number of internalized bacteria (CFU0) was determined. In the other wells, incubate the cells for another 6 h, add 0.25% Triton‐X‐100 sterile water to lyse the cells, and calculate the number of bacteria (CFU2) in the sterile water. The intracellular bactericidal activity of macrophages is expressed as (CFU0−CFU2)/CFU0×100%.

### Data and Statistical Analysis

Data were presented as the mean ± standard deviation (SD). Graphs and statistical analyses were performed using GraphPad Prism 8 software. The Shapiro–Wilk test was used to assess data distribution normality. The F‐test assessed the equality of variances between the two groups, while the paired t‐test with unequal variances was applied to compare the two groups. The Brown–Forsythe test evaluated variance homogeneity across multiple groups, and one‐way ANOVA was used to determine statistical differences among multiple groups. ^*^ indicates *p* < 0.05, representing a significant difference between groups; ^**^ indicates *p* < 0.01, representing a highly significant difference between groups.

## Conflict of Interest

The authors declare no conflict of interest.

## Author Contributions

X.R., J.L., and S.F. performed conceptualization. W.G. and S.F. performed resources. X.R., K.L., Y.L., W.G., and X.W. performed the methodology. X.R., B.Y., and S.F. performed visualization. B.Y. and J.L. performed supervision. X.R. and K.L. wrote the original draft. X.R., J.L., and S.F. did review & editing.

## Supporting information



Supporting Information

## Data Availability

The data that support the findings of this study are available from the corresponding author upon reasonable request.
