# Supplementary material for: HCAR2 Modulates the Crosstalk between Mammary Epithelial Cells and Macrophages to Mitigate Staphylococcus aureus Infection in the Mouse Mammary Gland
Source: Adv Sci (Weinh). 2025 Jan 10;12(9):2411947. doi: 10.1002/advs.202411947 (PMC11884543; doi:10.1002/advs.202411947)
Supplement: Supplementary file 1 — Supporting Information [file ADVS-12-2411947-s001.docx]

Table S1. The information of antibody in this study

| antibody | company | Catalog number |
| --- | --- | --- |
| GSDMD | Cell Signaling Technology | 69469S |
| HCAR2 | Novusbio | 92180 |
| N-GSDMD | Cell Signaling Technology | 36425S |
| NLRP3 | Cell Signaling Technology | 13158S |
| ASC | Proteintech | 67494-1-Ig |
| Caspase-1 | Proteintech | 22915-1-AP |
| ZO-1 | Cell Signaling Technology | 13663S |
| Occludin | Cell Signaling Technology | 91131S |
| Claudin3 | Cell Signaling Technology | 83609S |
| CMPK2 | Proteintech | 25877-1-AP |
| cGAS | Proteintech | 29958-1-AP |
| STING | Proteintech | 80165-1-RR |
| p-MLKL | Cell Signaling Technology | 37333S |
| MLKL | Cell Signaling Technology | 37705S |
| CD11b-APC | Biolegend | 101212 |
| F4/80-FITC | Biolegend | 123108 |
| Ly-6G-FITC | Biolegend | 127606 |
| F4/80 | Cell Signaling Technology | 30325S |
| dsDNA | PROGEN | AC-30-10 |
| β-actin | Proteintech | 66009-1-Ig |
| goat anti-rabbit | BOSTER Biological Technology | BA1055 |
| goat anti-mouse | BOSTER Biological Technology | BA1051 |

Table S2. Primers Used for qRT-PCR

| Gene | Sequences | Sequence length |
| --- | --- | --- |
| *NLRP3* | F: GAGCTGGACCTCAGTGACAATGC | 146 bp |
|  | R: ACCAATGCGAGATCCTGACAACAC |  |
| *CMPK2* | F: GGCAATTATCTCGTGGCTTC | 101 bp |
|  | R: GTAGCTATGGCGTAGGTGGC |  |
| *Mefv* | F: AGGCTTCAAGGACTTTACAACAA | 196 bp |
|  | R: TCATGCGAATGAGACTCCCAT |  |
| *Irf1* | F: ATGCCAATCACTCGAATGCG | 203 bp |
|  | R: CCTGCTTTGTATCGGCCTGT |  |
| *TLR2* | F: TCTAAAGTCGATCCGCGACAT | 155 bp |
|  | R: CTACGGGCAGTGGTGAAAACT |  |
| *Ncf1* | F: GTGGTCTACAGAAAATTCACCGA | 193 bp |
|  | R: CCATGAGGCCGTTGAAGTATTC |  |
| *β-actin* | F: GTCAGGTCATCACTATCGGCAAT | 147 bp |
|  | R: AGAGGTCTTTACGGATGTCAACGT |  |
| *Il-1β* | F: GTTCCCATTAGACAACTGCACTACA | 162 bp |
|  | R: GTCGTTGCTTGGTTCTCCTTGTAA |  |
| *IL-6* | F: CCAGAAACCGCTATGAAGTTCC | 138 bp |
|  | R: GTTGGGAGTGGTATCCTCTGTGA |  |
| *TNF-α* | F: CCCCAAAGGGATGAGAAGTTC | 136 bp |
|  | R: CCTCCACTTGGTGGTTTGCT |  |
| *Occludin* | F: ACACTTGCTTGGGACAGAGG | 197 bp |
|  | R: AAGGAAGCGATGAAGCAGAA |  |
| *Claudin3* | F: ACCAACTGCGTACAAGACGAG | 148 bp |
|  | R: CGGGCACCAACGGGTTATAG |  |
| *ZO-1* | F: GACCTTGATTTGCATGACGA | 199 bp |
|  | R: AGGACCGTGTAATGGCAGAC |  |
| *D-loop* | F: AATCTACCATCCTCCGTGAAACC | 90 bp |
|  | R: TCAGTTTAGCTACCCCCAAGTTTAA |  |
| *ND2* | F: CCCCATTCCACTTCTGATTACC | 89 bp |
|  | R: GGGAGCAATTTTTTGTCATGTAAGA |  |
| *Tert* | F: CTAGCTCATGTGTCAAGACCCTCTT | 110 bp |
|  | R: GCCAGCACGTTTCTCTCGTT |  |

Table S3. The primer sequences

| **Gene** | **Sequences** |
| --- | --- |
| *siHCAR2* | F：AGGCAGAGACAGAUGGACATT  R：UGUCCAUCUGUCUCUGCCUTT |
| *siCMPK2* | F：ACUUUGAAUGUCUUCUGAUTT  R：AUCAGAAGACAUUCAAAGUTT |
| *OE-CMPK2* | F: GGGTCAATATGTAATTTTCAGTG |
|  | R: CCTTATAGTCCTTATCATCGTC |

**Supplementary Figure S1**


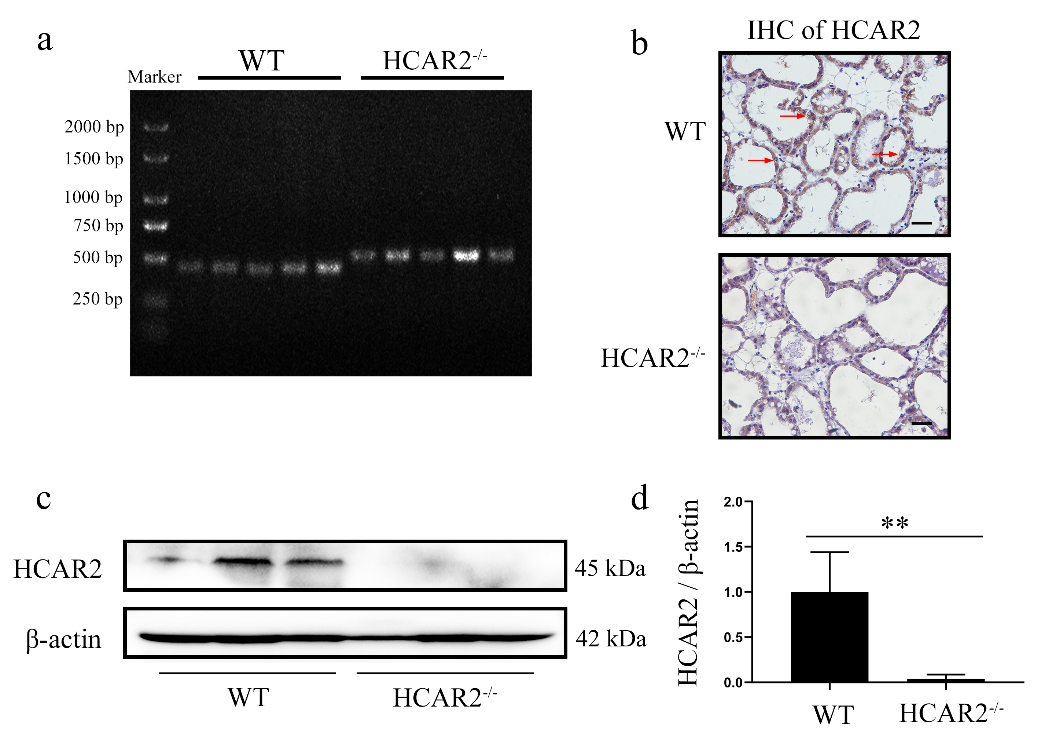


(a) Agarose gel electrophoresis was employed to analyze the genotypes of WT and HCAR2^-/-^ mice, n=5. (b) IHC of HCAR2 in mammary tissue, red arrows indicate HCAR2, scale bar represents 50 μm. (c, d) Western blot analysis of HCAR2 protein expression in mammary tissue. Results are presented as mean ± SD, n = 3.

**Supplementary Figure S2**


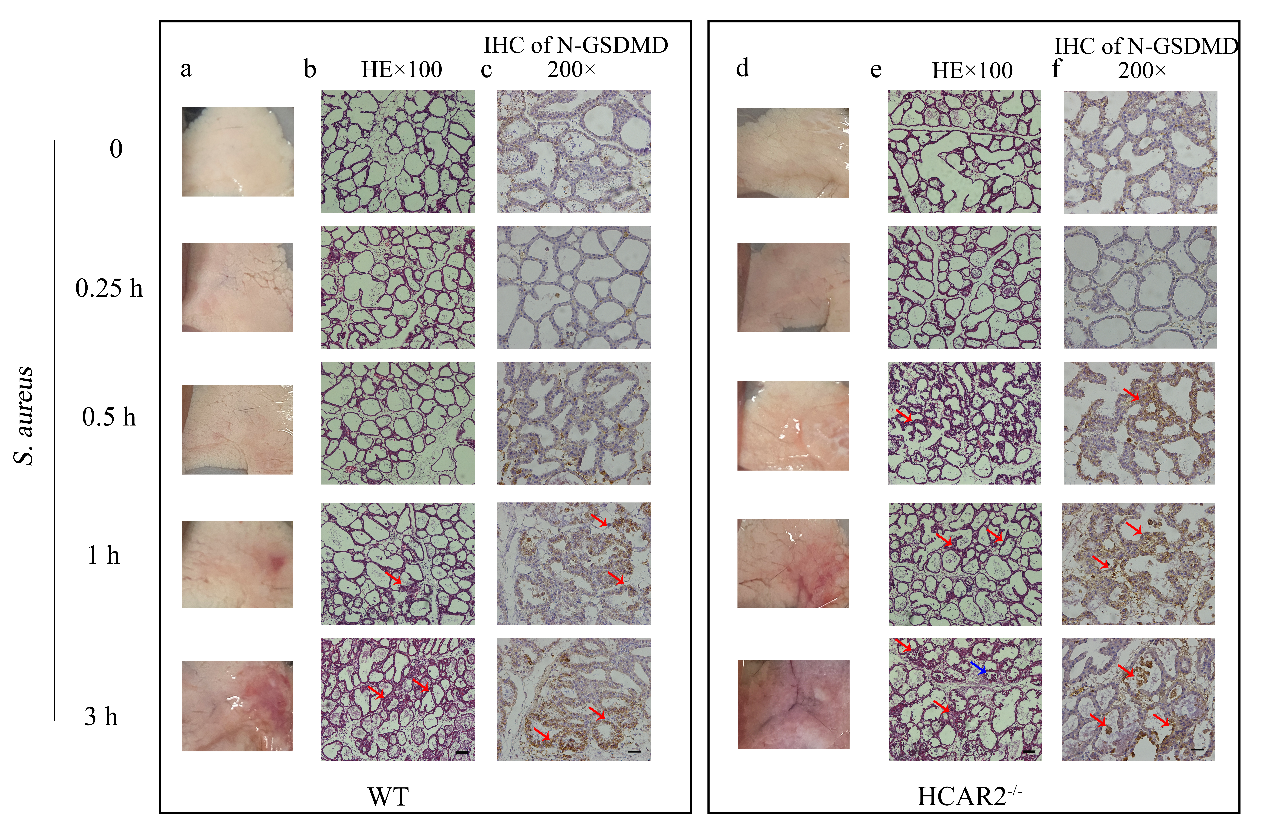


WT mice and HCAR2^-/-^ mice as experimental subjects, we harvested mammary tissues from the mammary ducts following a 0.25 h, 0.5 h, 1 h, and 3 h infusion of *S. aureus*. (a, b, d, e) Images of mouse mammary glands and H&E staining; red arrows highlight thickened acinar walls, blue arrow inflammatory cell infiltration, and scale bar represents 100 μm. (c, f) IHC of N-GSDMD in mammary tissue, scale bar represents 50 μm. Results are presented as mean ± SD, n = 6.

**Supplementary Figure S3**


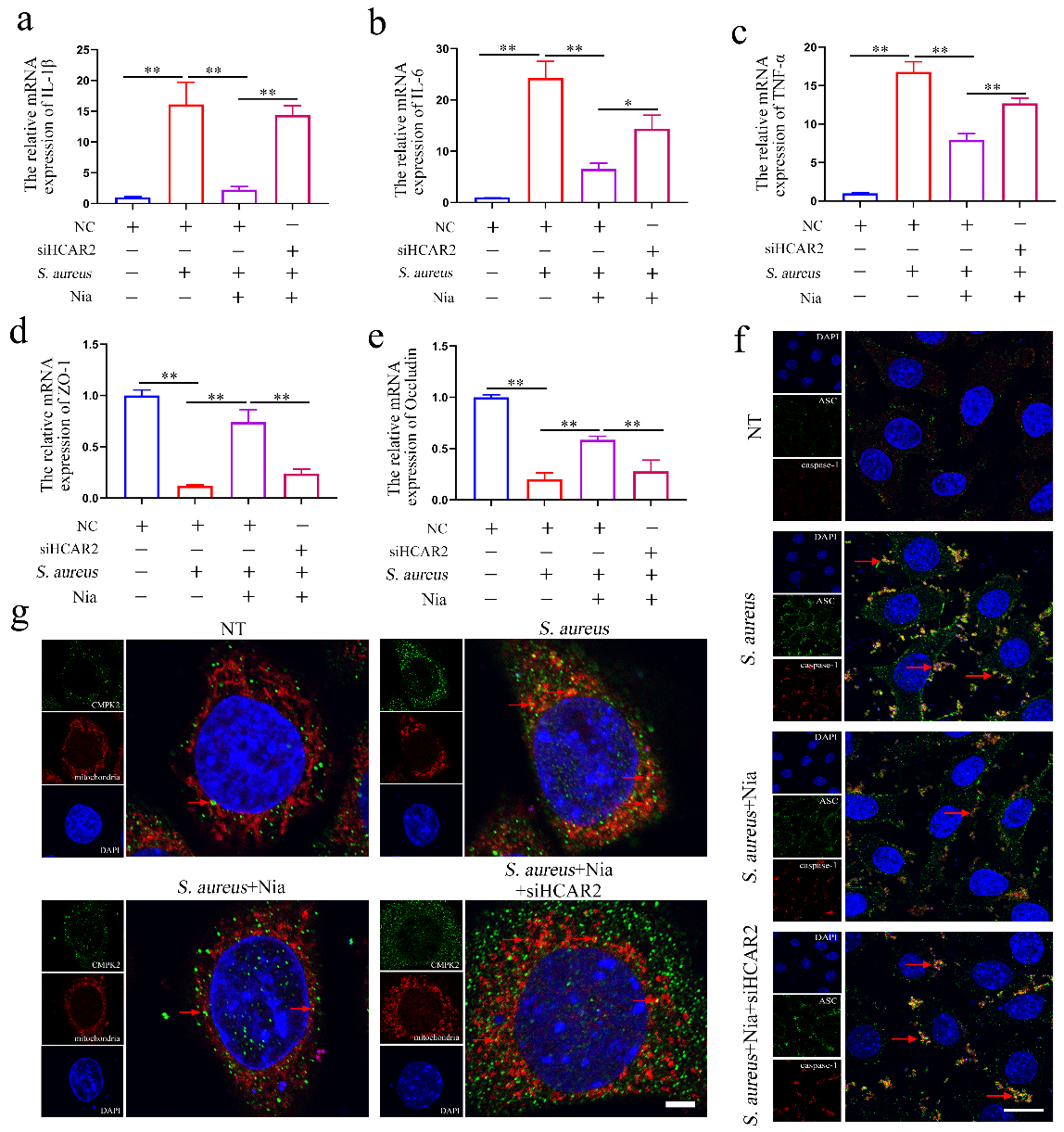


(a-e) qRT-PCR was used to measure mRNA levels of IL-1β, IL-6, TNF-α, ZO-1 and Occludin. (f) Immunofluorescence of ASC and caspase-1 within mMECs, using a laser confocal microscope for imaging. The red arrow indicates co-localization of ASC and caspase-1, and the scale represents a magnification of 5 μm. (g) CMPK2 antibodies and mitotracker were used to label CMPK2 and mitochondria, which were then imaged using a laser confocal microscope. Red arrows indicate co-localization of CMPK2 and mitochondria, with a scale bar of 2 μm. Results are presented as mean ± SD, n = 3.

**Supplementary Figure S4**


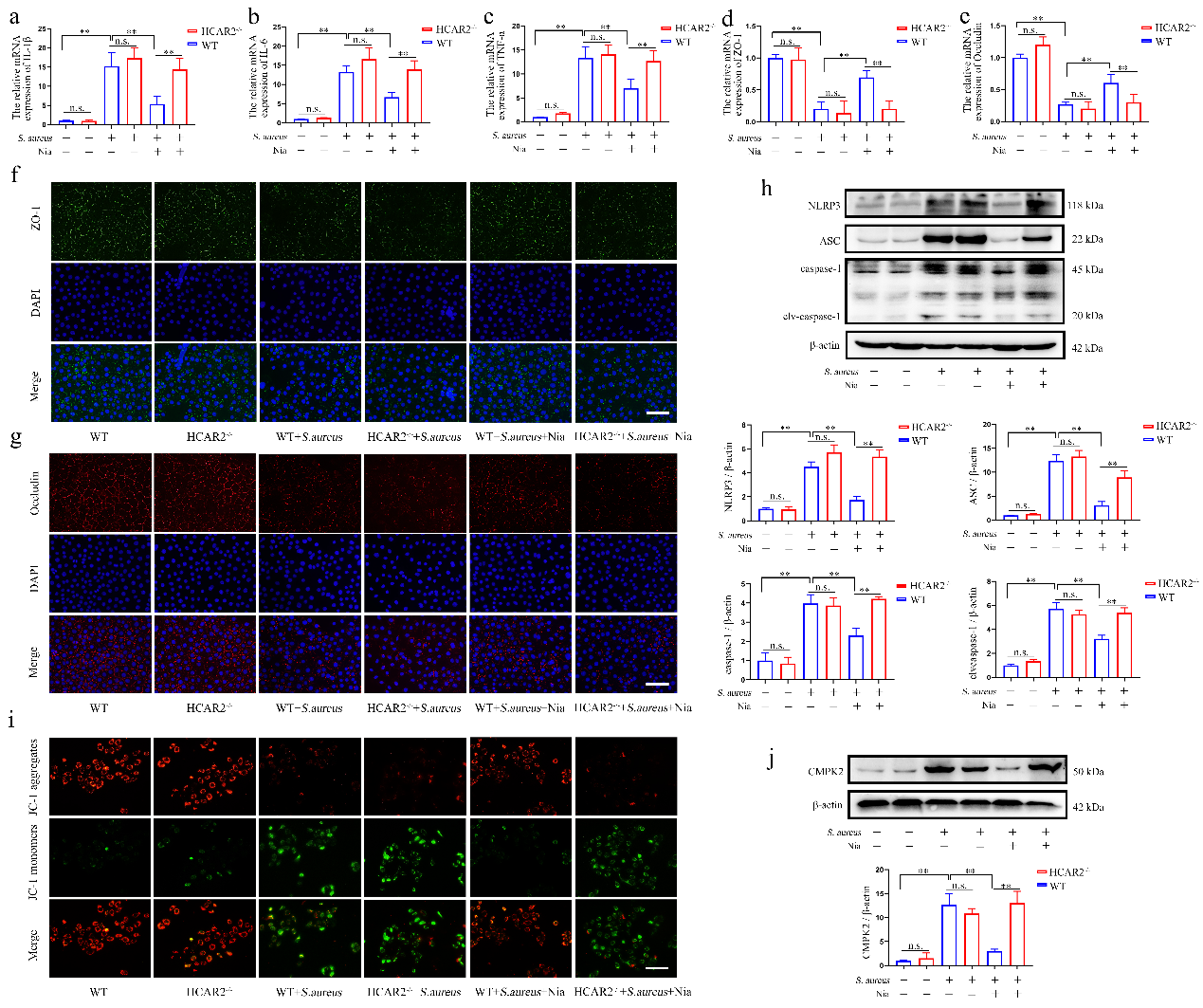


(a-e) qRT-PCR is used to detect mRNA levels of IL-1β, IL-6, TNF-α, ZO-1 and Occludin in in primary mMEC. (f, g) Immunofluorescence of ZO-1 and Occludin in primary mMEC. Scale represents 100 μm. (h) Western Blot is used to detect the protein expression of NLRP3, ASC and caspase-1 in primary mMEC. (i) The mitochondrial membrane potential of primary mMEC was assessed using the JC-1 membrane potential assay kit, with a scale bar of 100 μm. (j) Western Blot is used to detect the protein expression of CMPK2 in primary mMEC. Results are presented as mean ± SD, n = 3.

**Supplementary Figure S5**


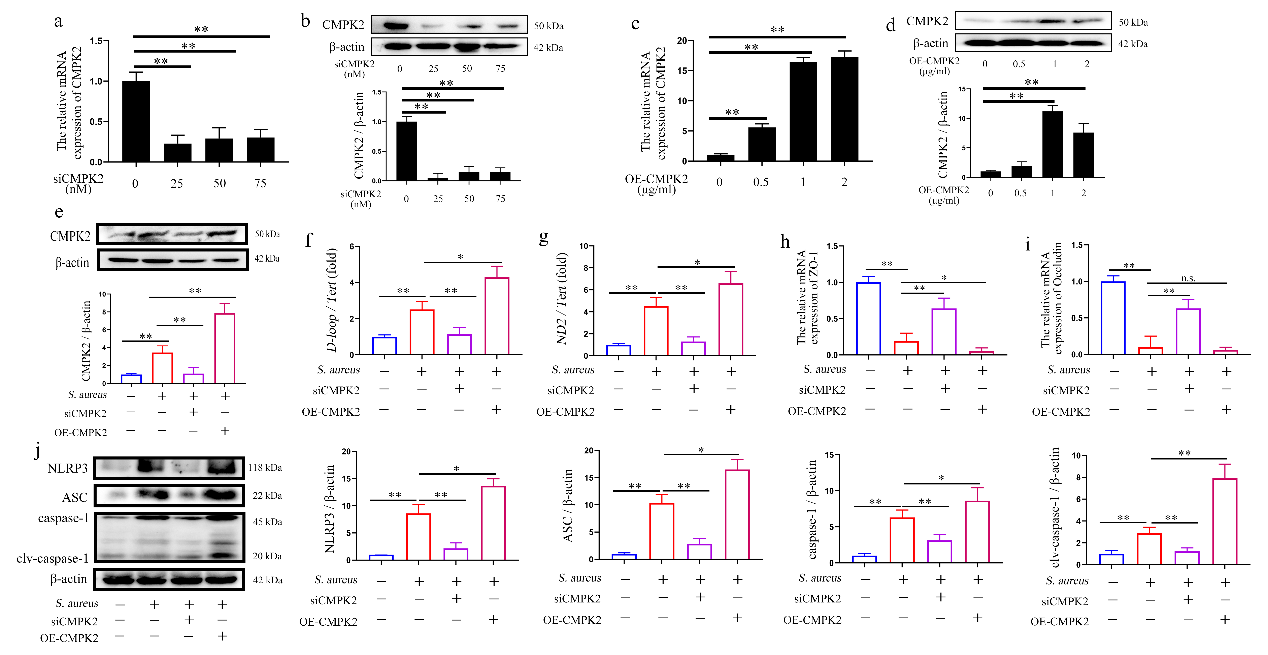


(a, c) qRT-PCR was used to measure mRNA levels of CMPK2 in mMECs. (b, d, e) Western blot analysis was used to measure CMPK2 protein expression in mMECs. (f, g) qPCR was performed to detect specific primers for mtDNA (D-loop, ND2) and nDNA (Tert), and to determine the relative content of mtDNA. (h, i) qRT-PCR was used to measure mRNA levels of ZO-1 and Occludin. (j) Western blot analysis was used to measure of NLRP3, ASC and caspase-1 proteins expression in mMECs. Results are presented as mean ± SD, n = 3.

**Supplementary Figure S6**


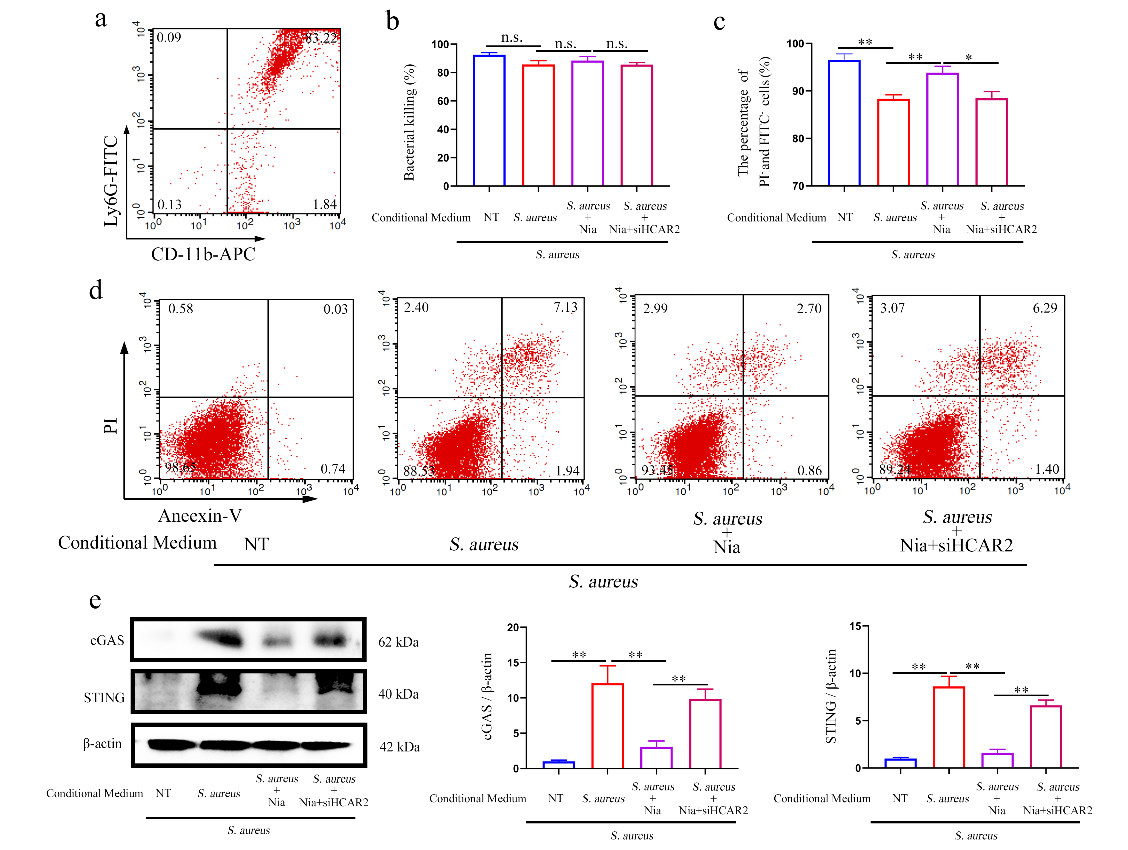


(a) The Purity of primary abdominal neutrophils was assessed using Ly6G and CD11b labeling. After 6-h incubation with supernatants from various mMEC groups, neutrophils were subsequently infected with *S. aureus* for an additional 6 h. (b) Neutrophils bactericidal activity against *S. aureus* was measured by plate counting. (c) The proportion of viable neutrophils was determined in each group. (d) Flow cytometry was performed to assess cell viability. (e) Western blot analysis was used to detect the protein expression of cGAS and STING. Results are expressed as mean ± SD, n = 3.

**Supplementary Figure S7**


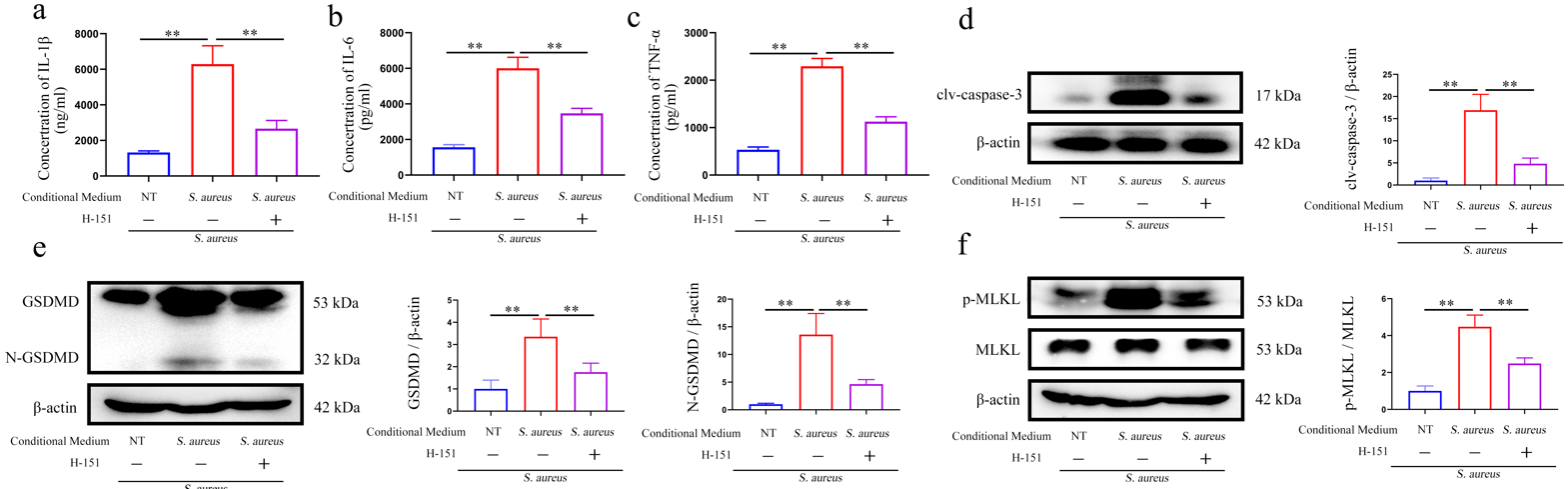


(a-c) ELISA was employed to quantify IL-1β, IL-6, and TNF-α levels in the macrophage culture supernatant. (d-f) Western blot analysis was used to detect the protein expression of cleaved caspase-1, p-MLKL, MLKL, and GSDMD. Results are expressed as mean ± SD, n = 6.

**Supplementary Figure S8**


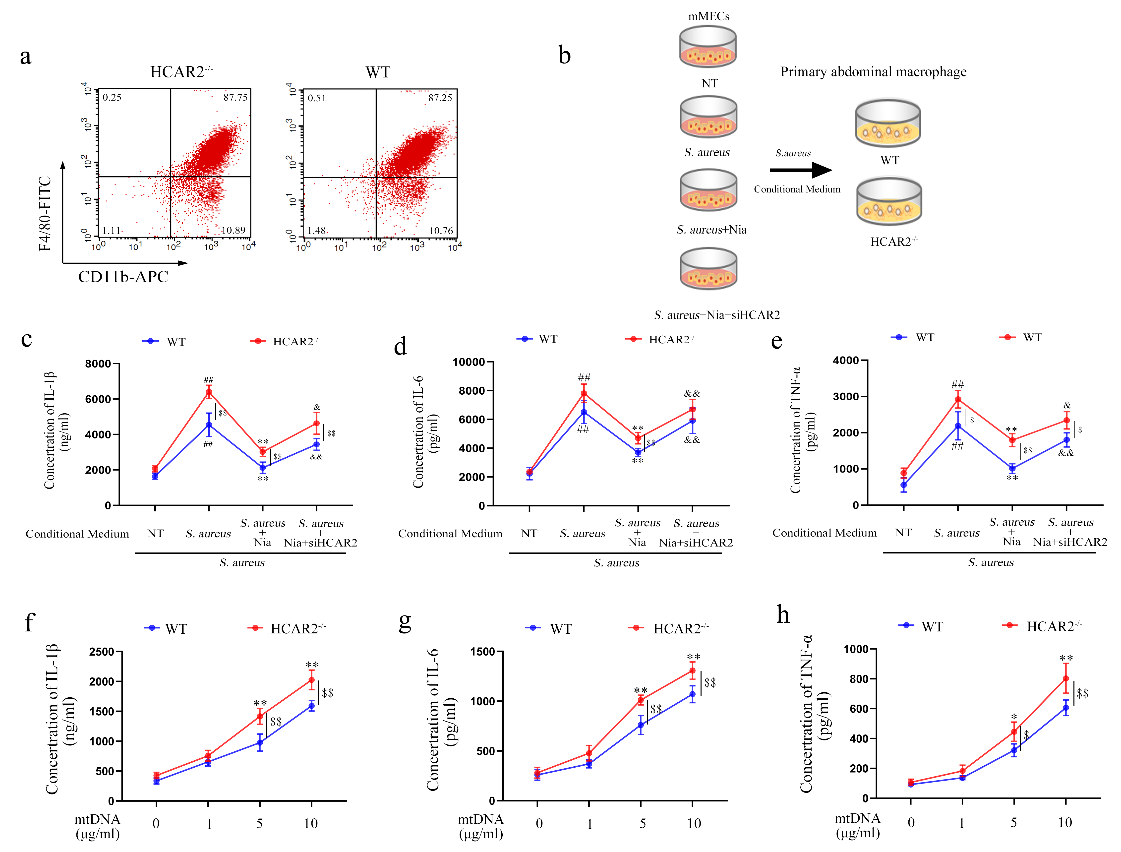


(a) Purity of primary peritoneal macrophages was assessed using F4/80 and CD11b labeling. (b) A schematic representation illustrating the co-stimulation of macrophages by supernatants from mMECs in conjunction with *S. aureus*. (c-h) ELISA was employed to quantify of IL-1β (c, f), IL-6 (d, g), and TNF-α (e, h) levels in the macrophage culture supernatant. Statistical significance is indicated as follows: ## p < 0.01 compared to the NT group; * and ** p < 0.05 and p < 0.01, respectively, compared to the S. aureus group; & and && p < 0.05 and p < 0.01, respectively, compared to the S. aureus + Nia group; $ and $$ p < 0.05 and p < 0.01, respectively, compared to WT mice. Results are expressed as mean ± SD, n = 3.

**Supplementary Figure S9**


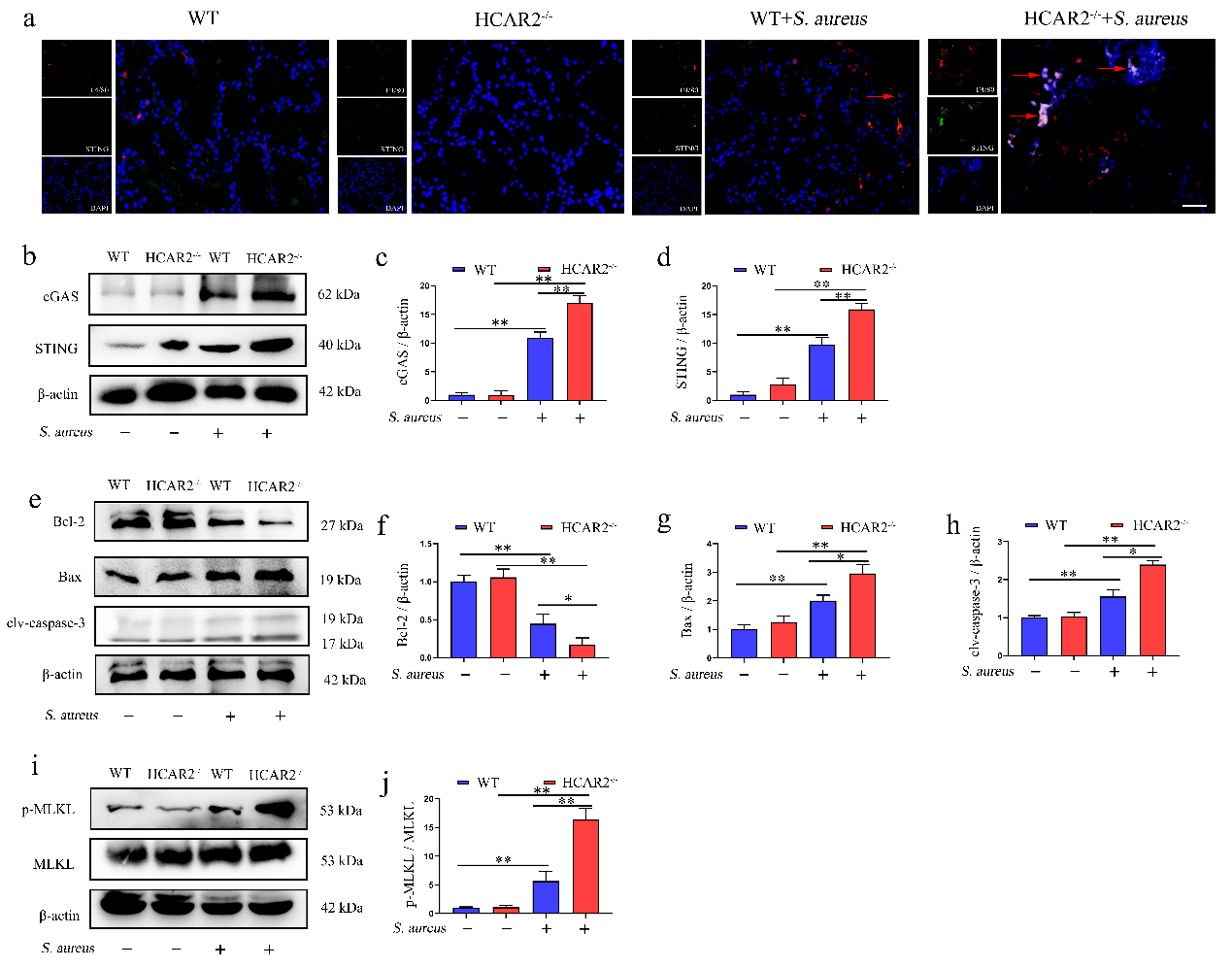


(a) Immunofluorescence staining of F4/80 and STING in mammary tissue, indicated by, Red arrows indicate co-localization of F4/80 and STING, with a scale bar of 20 μm. (b) Western blot analysis of cGAS and STING protein expression in mammary tissue. (c) Western blot analysis of Bcl2, Bax, and clv-caspase-3 protein expression in mammary tissue. (d) Western blot analysis of p-MLKL and MLKL protein expression in mammary tissue. Results are presented as mean ± SD, n = 6.

**Supplementary Figure S10**


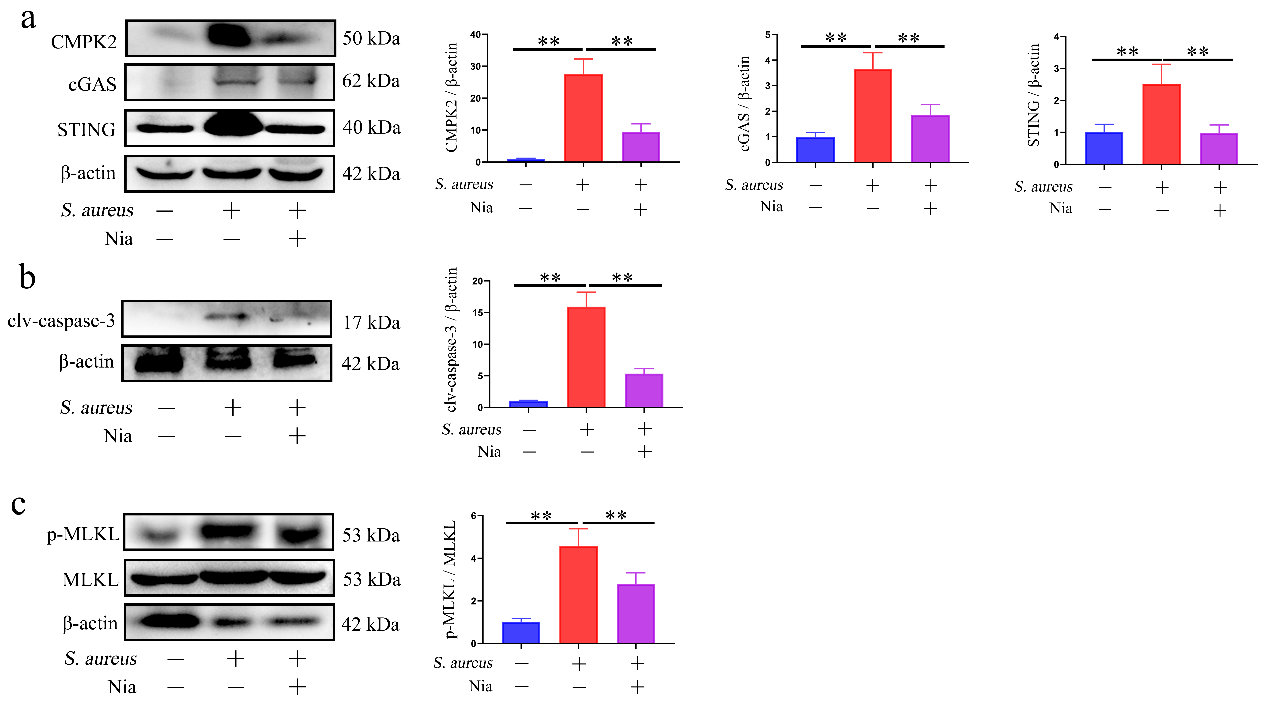


(a) Western blot analysis of CMPK2, cGAS, and STING protein expression in mammary tissue. (b) Western blot analysis of clv-caspase-3 protein expression in mammary tissue. (c) Western blot analysis of p-MLKL and MLKL protein expression in mammary tissue. Results are presented as mean ± SD, n = 6.
